# Impact of deep learning on CT-based organ-at-risk delineation for flank irradiation in paediatric renal tumours: a SIOP-RTSG radiotherapy committee study

**DOI:** 10.1016/j.ctro.2025.101051

**Published:** 2025-09-19

**Authors:** Mianyong Ding, Matteo Maspero, Semi Harrabi, Emmanuel Jouglar, Sabina Vennarini, Timothy Spencer, Britta Weber, Henriette Magelssen, Karen Van Beek, Remus Stoica, Simonetta Saldi, Tom Boterberg, Patrick Melchior, Marry M. van den Heuvel-Eibrink, Geert O. Janssens

**Affiliations:** a Princess Máxima Centre for Paediatric Oncology, Utrecht, Netherlands (the); b Department of Radiation Oncology, Imaging and Cancer Division, University Medical Centre Utrecht, Utrecht, Netherlands (the); c Computational Imaging Group for MR Diagnostics & Therapy, Centre for Image Sciences, University Medical Centre Utrecht, Utrecht, Netherlands (the); d University Hospital Heidelberg, Heidelberg Ion Beam Therapy Centre (HIT), Dept. of Radiation Oncology, Heidelberg, Germany; e Department of Radiation Oncology, Institut Curie, PSL University, Paris, France; f Paediatric Radiotherapy Unit, IRCCS Fondazione Istituto Nazionale Tumori-Milan, Italy; g Bristol Cancer Institute, Bristol, UK; h Danish Centre for Particle Therapy, Aarhus, Denmark; i Department of Oncology, Oslo University Hospital, Oslo, Norway; j Department of Radiation Oncology, University Hospital Leuven, Belgium; k Emergency Clinical Hospital for Children “Marie Skłodowska Curie”, Bucharest, Romania; l Hospital Santa Maria della Misericordia, Perugia, Italy; m Department of Radiation Oncology, Ghent University Hospital, Ghent, Belgium; n Department of Radiation Oncology, University of Saarland, Homburg, Germany; o Wilhelmina Children’s Hospital-Division of CHILD HEALTH, University Medical Centre Utrecht, University of Utrecht, Utrecht, Netherlands (the)

**Keywords:** Artificial intelligence, Auto-contouring, Inter-observer variability, Wilms tumors, Flank irradiation, Organs-at-risk

## Abstract

**Background and purpose:**

Integrating deep learning (DL) for auto-contouring has significantly improved organ-at-risk (OAR) delineation in adult radiotherapy. However, its application in paediatric radiotherapy remains limited. This study evaluates DL-based auto-contouring of OARs, followed by manual revisions, for paediatric flank irradiation, focusing on delineation time, accuracy, and inter-observer variability (IOV).

**Materials and methods:**

Twelve paediatric radiation oncologists from nine countries affiliated with the SIOP Renal Tumour Study Group participated in a two-day workshop. Participants were randomly divided into two groups: one performed manual delineation first, followed by DL-based revision, while the other group performed in reverse order. Eight thoracoabdominal OARs were delineated on non-contrast CTs of renal tumour patients (ages 1–6). DL-based contours were generated using a model for paediatric abdominal cases. Delineation time was recorded, accuracy and IOV were assessed using the Dice similarity coefficient (DSC), 95th percentile Hausdorff distance, mean surface distance against a STAPLE consensus (threshold = 0.95), and an expert reference.

**Results:**

In total, 122 manual delineations and 254 DL-based revisions were collected. DL-based auto-contouring reduced delineation time by 59 %, from 25.5 to 10.2 min. The mean DSC of all eight OARs improved from 0.91 to 0.97 using STAPLE reference and from 0.89 to 0.93 using expert reference. The pancreas exhibited the largest gain, with mean DSC increases ranging from 0.18 to 0.25. Delineation accuracy was significantly improved for seven OARs (p < 0.05), while IOV significantly decreased for the pancreas and heart in both references (p < 0.05).

**Conclusion:**

Manually revising DL-based auto-contouring reduces delineation time, enhances accuracy, and reduces inter-observer variability in paediatric CT-based OAR delineation.

## Highlights

• Evaluation of models for non-CNS OAR delineation in paediatrics is underexplored.
• Twelve observers validated DL-based auto-contouring of OARs applied in flank RT.
• DL-based OAR auto-contouring reduced the delineation time by 59 %.
• DL-based OAR auto-contouring improved the accuracy of delineation.
• Inter-observer variability of OAR delineation was reduced using auto-contouring.

## Introduction

Renal tumours are among the most common paediatric upper abdominal malignancies [1], with over 20 % of paediatric patients requiring post-operative flank irradiation [2]. To mitigate the risk of radiotherapy-induced late effects, the International Society for Paediatric Oncology–Renal Tumour Study Group (SIOP-RTSG) has recently developed a consensus statement on highly conformal flank target volume delineation [3]. This systematic approach, adjusting for organ shifts in the surgical cavity, enhances the sparing of surrounding organs-at-risk (OARs) when combined with modern radiotherapy modalities [3,4].

Manual OAR delineation is time-consuming [5] and prone to inter-observer variability (IOV) [6], which can affect the dose given to the organs [7] and potentially clinical outcomes. Deep learning (DL) models have emerged as a promising solution for automatic delineation. Numerous studies have focused on developing auto-segmentation models on computed tomography (CT), demonstrating good accuracy and clinical acceptability [8,9]. With human revision, DL-based models in adults significantly reduce delineation time compared to manual contouring [5,8,[10], [11], [12]]. Additionally, these models reduce IOV and produce more consistent contours [[12], [13], [14]]. However, most studies have been developed and validated using adult-specific datasets [5,[8], [9], [10], [11], [12], [13], [14]], with a limited focus on paediatric settings [[15], [16], [17], [18]].

Paediatric patients pose unique challenges for auto-segmentation due to the limited availability of datasets and substantial organ variability across different age groups. Models trained solely on adult datasets may underperform on paediatric data, as demonstrated by studies that compared adult and paediatric abdominal OARs [17,18]. Commercial auto-contouring software, which is generally developed using adult datasets, has demonstrated reduced performance in paediatric patients [19]. This highlights the need to develop and critically evaluate paediatric-specific auto-segmentation models.

Designing paediatric-specific auto-contouring models can yield more accurate segmentation results for children than adult-exclusive models in paediatric settings [17,18]. To our knowledge, only a few studies have investigated DL-based paediatric-specific models for OAR delineation in paediatric patients, primarily focusing on the brain [20] and thoracoabdominal regions [[16], [17], [18],^21^,^22^]. Despite these developments, no study has thoroughly evaluated a deep learning-based auto-contouring model designed explicitly for paediatric abdominal OARs in a clinical setting.

In our previous work, we developed and validated a CT-based DL auto-segmentation model for 17 OARs in paediatric patients with upper abdominal tumours, showing promising clinical usability [16]. Building on these findings, we organised a workshop with the SIOP-RTSG Radiotherapy Committee members to investigate the impact of DL on CT-based OAR delineation for flank irradiation in paediatric renal tumours. Before the workshop, we conducted a survey to gather information on patient care and imaging practices at the participants' centres. During the workshop, we assessed the model's impact on three key aspects: (1) time savings, (2) delineation accuracy, and (3) inter-observer variability (IOV).

## Materials and methods

### Participants and pre-workshop survey

Twelve international SIOP-RTSG panel members in paediatric radiation oncology from twelve medical centres across nine European countries participated in this study. The institutions are located in Belgium (2), Germany (2), Italy (2), Denmark (1), France (1), the Netherlands (1), Norway (1), Romania (1), and the United Kingdom (1).

Before the live workshop, a survey was distributed to the participants to assess how patient care and imaging practices were applied at their centres. The survey consisted of 20 questions (Supplementary Material 1), a part of which was adapted from a previous study [23] and addressed three main areas:

Participant experience: professional background in radiation oncology and the number of paediatric patients irradiated within the department annually.
Perspectives on artificial intelligence (AI): views on AI-based auto-contouring tools' impact and potential risks in radiotherapy.
Imaging and contouring practices: details on imaging modalities, e.g., MRI usage, contrast application, and contouring software currently in use, along with the implementation of auto-contouring for OAR delineation.

### Patient data and imaging protocol

This study involved paediatric patients with renal tumours diagnosed at the Princess Máxima Centre undergoing flank irradiation at the University Medical Centre Utrecht. The patients were randomly selected from the test dataset of a previous study [16], independent of the model’s training dataset and without consideration of tumour side. To ensure a representative cohort in terms of patient age and anatomical variation, ten patients aged 1–6 years who had undergone surgery with nephrectomy were included. The selected cases were representative of the average model performance and did not include instances of extremely low performance or failed predictions. CTs were acquired using a BigBore CT (Brilliance, Philips Medical Systems, Best, the Netherlands) with a consistent slice thickness of 2.0 mm and pixel spacing ranging from 0.8 to 1.4 mm. All imaging was performed without the use of intravenous or oral contrast.

### DL-based auto-contouring

A previously developed and open-source^1^ deep learning-based auto-contouring model was evaluated for thoracoabdominal OARs [16]. The model employed a self-configured nnU-Net framework with a 3D full-resolution U-Net architecture [24]. It was developed based on a combined dataset comprising 189 in-house CTs from paediatric patients with renal tumours and abdominal neuroblastoma, sharing the exact OAR definition. Additionally, it included 189 paediatric CTs from an open-access dataset without specified pathology but covering similar anatomical regions [25]. Although the original model could segment seventeen OARs, this study focused on nine commonly delineated organs: heart, spleen, liver, lungs (left and right), kidneys (left and right), pancreas, and stomach-bowel.

### Study design

The study was conducted as a two-day live workshop in Utrecht, the Netherlands, in November 2024, using the browser-based contouring platform ProKnow (v2.0.2.0, Elekta AB, Stockholm, Sweden). Recommended window levels and delineation guidelines for each OAR were provided to assist participants and to ensure consistency (Supplementary Material 2). Additionally, the slice interpolation and brush functions are accessible through ProKnow. Before starting or revising the DL-made delineation, participants completed a 30-minute exercise to familiarise themselves with the software. During the contouring session, all participants were provided with identical infrastructure, including the same type of PC, a single screen, and a standardised mouse and keyboard setup.

**Fig. 1:**
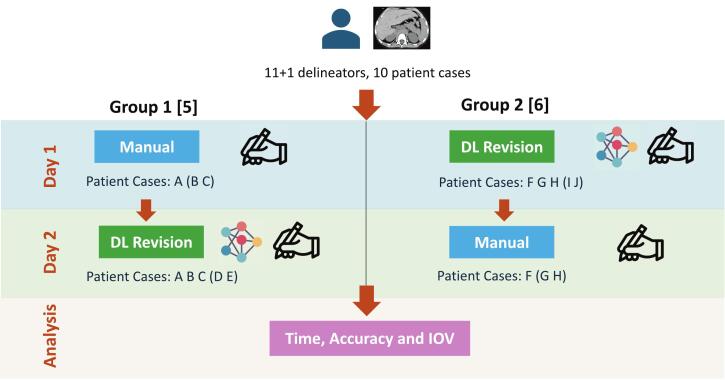
Workflow of the study. Numbers between brackets indicate the number of delineators per group. Each letter represents a different patient case, with cases between parentheses optional for participants. Twelve participants joined the study, with eleven participating in the two-day sessions as delineators and one manually providing the reference contours without time constraints.

Independent of the workshop, a senior radiation oncologist (GJ) manually delineated all cases to provide reference contours for comparison. The remaining eleven participants were divided into two groups, each completing two two-hour delineation sessions over two consecutive days, with ten patient cases evenly distributed over the groups (Fig. 1).

On the first day, one group revised DL-generated contours. At the same time, the other performed manual delineations from scratch, without support from the DL model, and with delineation interpolation allowed among slices. On the second day, the groups switched tasks to minimise potential biases related to software familiarity or memorisation of patient cases. Each participant was requested to complete a maximum of three patients during the manual delineation session and at least three patients during the DL revision session, with a maximum of five cases per participant. Participants within the same group were asked to follow the same patient order during the sessions.

Participants recorded the time spent on each organ for each patient during both sessions using an online browser-based stopwatch (https://vclock.com/stopwatch/). Contours from all participants across both sessions were collected via the ProKnow application programming interface. While incomplete patient structure sets were included in the analysis, incomplete organ structure delineations were excluded.

### Reference contours

Reference contours for each structure were created using single expert contours and consensus contours generated through simultaneous truth and performance level estimation (STAPLE) [26] with a threshold of 0.95.

In the single expert approach, a senior radiation oncologist (GJ) manually delineated each OAR without time constraints. The registered MRI was accessible during the reference-generating process to enhance the quality of the delineations.

The STAPLE approach generated consensus contours by combining all eleven participant contours. Two versions of STAPLE were created: (1) a combined STAPLE, which utilised all available contours from both sessions to create a single STAPLE reference for each structure of each patient, and (2) a session-specific STAPLE, which included only contours from a single session to generate separate STAPLE references for the Manual and DL revision sessions. For clarity, results from the session-specific STAPLE approach are reported exclusively in the Supplementary Material. In both STAPLE approaches, structures delineated by fewer than three participants were omitted from the computation. A comparison between the STAPLE and the single expert references can be found in Supplementary Material 3.

### Evaluation

All collected data were analysed to evaluate the impact of the DL model on performance in terms of delineation time, accuracy, and IOV. Since each patient had one left or right kidney, these structures were grouped for evaluation.

### Time

The reduction in time required for OAR delineation using the DL-based model compared to manual delineation was assessed. The time (in minutes) recorded for all OARs across both sessions was compared using the Wilcoxon rank-sum test to determine statistical significance.

### Accuracy and IOV

Delineation accuracy was evaluated by comparing participant-generated contours with reference contours using three metrics: the Dice similarity coefficient (DSC), the 95th percentile Hausdorff distance (HD95), and the mean surface distance (MSD). DSC measures volumetric overlap between participant and reference contours, HD95 evaluates the 95th percentile of surface distances to identify worst-case discrepancies, and MSD calculates the average boundary difference. The accuracy of manual and DL-based delineations was compared using the Wilcoxon rank-sum test for statistical significance.

IOV was quantified as the standard deviation (SD) of each structure's DSC, HD95, and MSD. A lower SD indicates greater consistency and lower IOV, while a higher SD reflects reduced consistency and higher IOV. Variance differences between manual and DL-based delineations were tested using Levene’s test for statistical significance.

In addition, other approaches to quantify IOV were conducted and are provided in the Supplementary Data. For example, pairwise comparisons of inter-observer variability using DSC, HD95, and MSD are provided in Supplementary Material 3. Furthermore, 3D surface variation analysis [27] was performed by calculating the local variation at each surface point and then averaging these values across all surface points of the OAR to obtain a global 3D SD, which is presented in Supplementary Data.

## Results

All twelve participants completed the survey, with 16 out of 21 questions receiving at least 11 responses (Supplementary Material 1). Participants had diverse levels of experience in radiation oncology, ranging from 2 to 25 years, with a median of 11 to 15 years of experience. All participants indicated that they treat fewer than 10 patients for flank irradiation annually in their departments, with seven reporting 1–5 cases annually. None of the participants had prior experience with the ProKnow software, starting at the same level of familiarity. At least six participants reported using CT with intravenous contrast and MRI for co-registration in clinical practice. In terms of implementation, five participants reported using AI-based auto-contouring followed by manual revision for OAR delineation in their daily practice. Eight participants indicated that their departments have integrated auto-contouring tools for OAR delineation in adults and paediatrics.

In total, 122 OARs were fully contoured during the manual delineation session, while the DL-based revision session yielded 254 OARs. The number of organs delineated per participant ranged from 6 to 24 (median, 10) in the manual session and from 7 to 32 (median, 24) in the DL-based revision session.

**Fig. 2:**
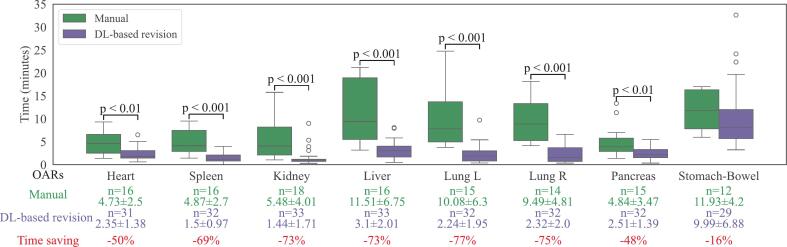
Comparison of delineation time per organ (in minutes) between manual contouring and deep learning (DL)-based revision. The green, purple, and red rows display the number of manual delineations (n) with the mean ± SD of manual delineation time, the time spent on DL-based revision, and the percentage of time savings (manual vs. DL-based revision), respectively. Only p-values below 0.05 are shown in the plot. (For interpretation of the references to colour in this figure legend, the reader is referred to the web version of this article.)

DL-based revision reduced mean delineation times compared to manual delineation for all evaluated OARs, with seven showing statistical significance (Fig. 2). In the manual session, the lungs, liver, and stomach-bowel had the longest mean times (9–12 min). With DL-based revision, liver and lung times decreased to 2–3 min—a reduction of over 70 %—while the stomach-bowel times showed a smaller decrease of 1.9 min (−16 %). Across all eight evaluated OARs, the mean delineation time decreased from 62.9 min for manual delineation to 25.5 min with DL-based revision, reflecting a 59 % reduction.

**Fig. 3:**
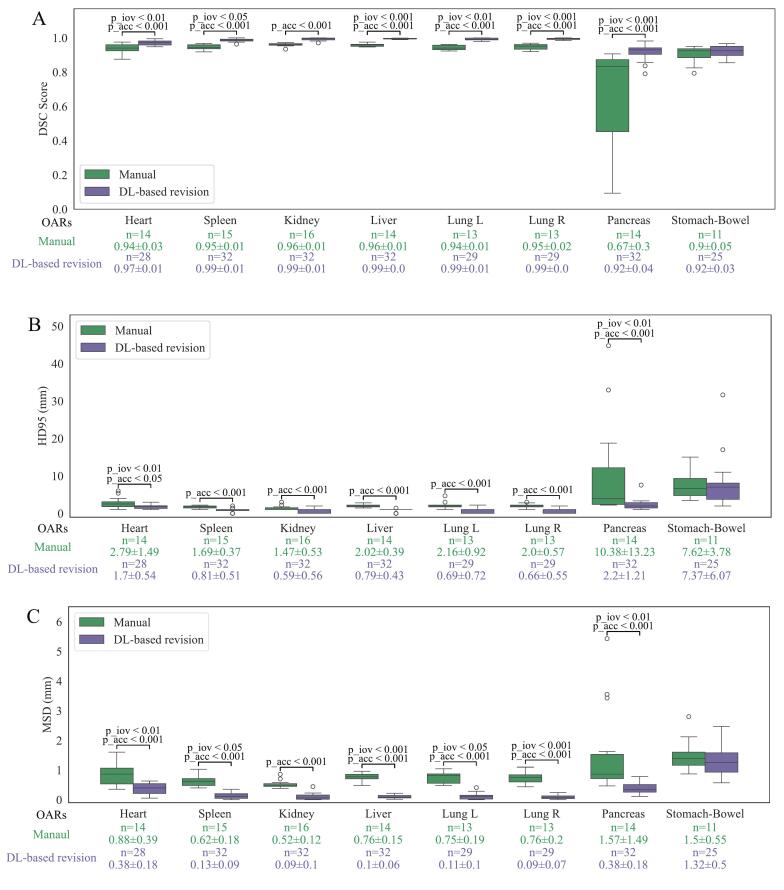
Accuracy and IOV of manual delineation and DL-based revision, measured by DSC(A), HD95(B), and MSD(C), performed by 11 delineators using STAPLE as the reference. The p_acc represents the Wilcoxon signed-rank test used to compare accuracy, while p_iov refers to Levene's test for homogeneity of variance. Only p-values smaller than 0.05 are displayed in the figure. The ‘green’ row shows the number of included delineations (n) and the mean ± standard deviation for manual delineation. The ‘purple’ row provides the same for DL-based revision. Structures delineated by fewer than three participants are excluded due to missing STAPLE references. (For interpretation of the references to colour in this figure legend, the reader is referred to the web version of this article.)

**Fig. 4:**
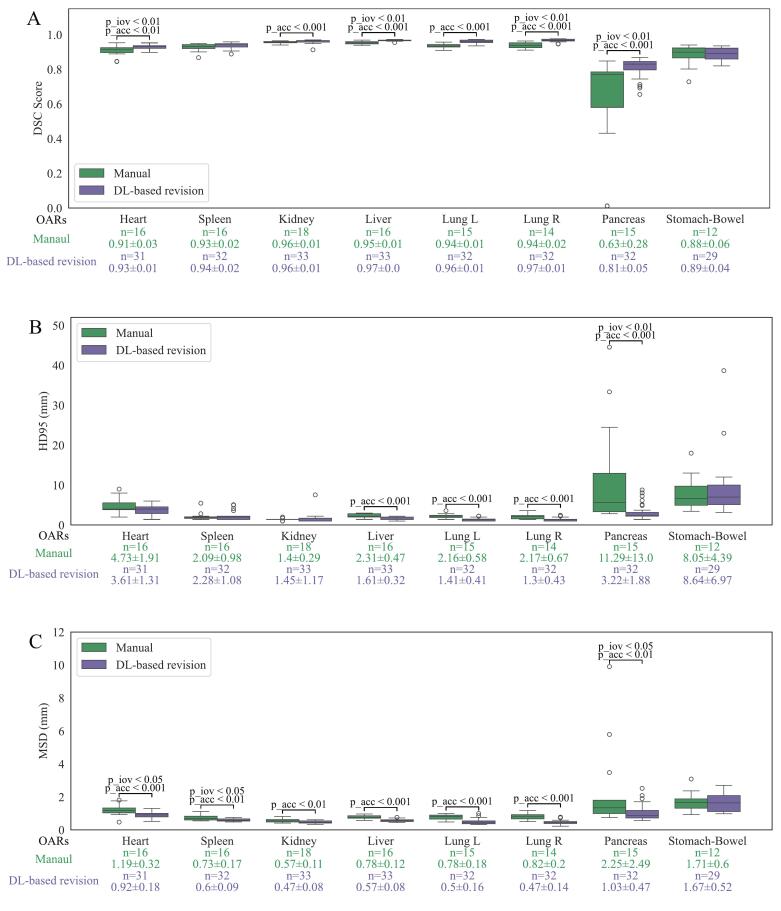
The accuracy and inter-observer variability (IOV) of manual delineation and DL-based revision, as measured by DSC (A), HD95 (B), and MSD (C), were assessed by 11 delineators using a single expert as a reference. The p_acc indicates the Wilcoxon signed-rank test used to compare accuracy, while p_iov represents Levene's test for homogeneity of variance. Only p-values smaller than 0.05 are displayed in the figure. The ‘green’ row shows the number of included delineations (n) and the mean ± standard deviation for manual delineation. The ‘purple’ row provides the same for DL-based revision. (For interpretation of the references to colour in this figure legend, the reader is referred to the web version of this article.)

Two types of reference contours were used to compare the accuracy and IOV of manual delineation and DL-based revision: STAPLE contours (Fig. 3) and single expert contours (Fig. 4).

For accuracy, using STAPLE contours as the reference, significant improvements (p < 0.05) were observed for 7 out of 8 OARs across all metrics, including higher DSC, lower HD95, and reduced MSD. The mean DSC for all OARs increased from 0.91 to 0.97, with significantly improved OARs showing DSC gains ranging from 0.03 to 0.25 after DL-based revisions. In comparison, using single expert contours as the reference showed fewer significant improvements, with the mean DSC increasing from 0.89 to 0.93. However, 7 out of 8 OARs demonstrated a significant enhancement in at least two metrics. Across both references, the pancreas showed the largest improvement, with the mean DSC rising from 0.67 to 0.92 using STAPLE contours and from 0.63 to 0.81 using single expert contours, while the stomach-bowel consistently showed no significant improvement.

**Fig. 5:**
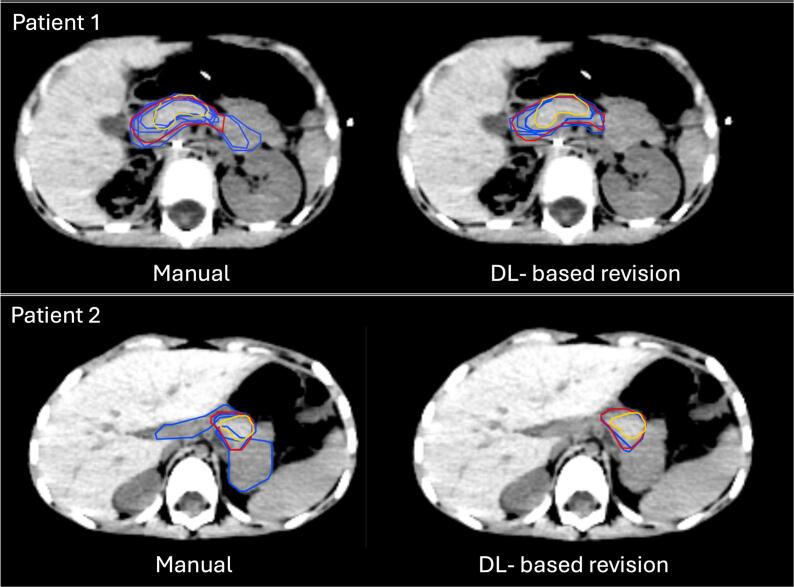
Examples of pancreas delineation variability in two patients. ‘Red’ contours are generated using STAPLE, while ‘yellow’ contours are expert-based, and each blue contour represents the delineation of one participant. The two patients are from two separate groups, with one patient delineated by 6 participants and one patient delineated by 5 participants. (For interpretation of the references to colour in this figure legend, the reader is referred to the web version of this article.)

Using STAPLE contours as the reference, standard deviations across DSC, HD95, and MSD were reduced or equal for all OARs across at least two evaluation metrics. Significant reductions in IOV (p < 0.05) were observed for the heart, spleen, liver, pancreas, and lungs in at least two metrics. With single-expert contours as the reference, significant reductions in IOV were noted only for the pancreas and heart, for at least two metrics. Pairwise comparisons showed significant reductions in IOV across all OARs (Supplementary Fig. 5). An example of the improvement in pancreas IOV is presented in Fig. 5.

## Discussion

This study compared manual delineation with DL-based revisions of OAR delineation in paediatric patients with a renal tumour undergoing flank irradiation to evaluate the impact on time efficiency, accuracy, and inter-observer variability. We observed that the DL-based approach reduced delineation time by approximately 60 %, improved delineation accuracy for 7 out of 8 OARs, and decreased IOV for at least 5 out of 8 OARs, considering STAPLE as a reference. These results underscore the effectiveness of including DL-based methods to streamline workflows and improve consistency in OAR delineation.

Our pre-workshop survey indicates that approximately 60 % of participating centres treat fewer than five paediatric renal tumour cases per year, underscoring the challenge of gathering sufficient data to develop robust, paediatric-specific models. Despite this limitation, all participants expressed positive perspectives on using DL-based auto-contouring, with over 75 % indicating that AI-based auto-contouring for OAR delineation has been implemented at their institutions for both adult and paediatric patients. This underscores the critical need for thorough, clinical, and standardised evaluation of AI-based auto-contouring in paediatric abdominal cases.

Only a few studies have clinically evaluated DL-based auto-contouring models for paediatric OARs in whole-body irradiation [19], craniospinal irradiation [28], and head and neck radiotherapy [29]. Our study addressed this gap by evaluating a paediatric-specific deep learning (DL) model tailored for OARs typically delineated for flank radiotherapy in Wilms tumours and abdominal neuroblastoma. Unlike our previous study, which relied on subjective scores from only two radiation oncologists [16] or other paediatric studies that assess accuracy against a reference without evaluation made by clinicians [17,18,20,21]. This study included a comprehensive quantitative clinical evaluation of the impact of DL on the delineations by a large group of paediatric radiation oncologists. Our findings align with adult-focused studies that reported 36–86 % time savings for thoracoabdominal organs [5,8,11], improved OAR accuracy, and reduced IOV for head-and-neck [12] and breast cancer cases [13]. To our knowledge, this is among the first studies to evaluate the time savings, accuracy, and IOV of deep learning-based segmentation in paediatric radiotherapy.

When assessing accuracy and IOV, we used two reference approaches. The single-expert approach benefits from access to MRI, enabling precise delineations without the influence of DL; however, it may be subject to individual biases. In contrast, the STAPLE method combines contours from all delineators to achieve a consensus, reducing individual bias and variability. However, STAPLE is influenced by varying participant expertise and incorporates AI-revised contours, which may introduce additional bias. In this study, we combined the two methods to mitigate the limitations of both methods. Our results showed significant accuracy improvements in 7 out of 8 OARs with both references. At the same time, IOV reductions varied depending on the reference method: five OARs showed significant IOV reductions with the STAPLE reference, whereas only two did so with the single-expert reference. These two complementary reference methods show that our DL-based approach impacts accuracy and inter-observer variability.

One limitation of this study was the restricted imaging modality, as imaging was limited to single-centre, non-contrast CTs without MRI co-registration. As noted in the responses from our survey, more than 60 % of the participants reported using CT with intravenous contrast and MRI for co-registration in clinical practice. The absence of contrast-enhanced CT and MRI in our study may have impacted the accuracy and IOV, especially for the pancreas, which is a notoriously challenging organ. However, it is worth noting that in case contrast-enhanced CT or MRI is available, the performance of the DL model may also vary, as shown in a previous study reporting an increase of DSC thanks to the use of contrast-enhanced CT [16]. Additionally, another study indicates that a multimodal model trained using co-registered MRI with CT has the potential to improve performance [30].

Another limitation of this study is that the DL revision and manual sessions were conducted on consecutive days with overlapping patients, making some memorization of anatomy inevitable. In addition, as all participants were unfamiliar with ProKnow, residual learning-curve effects may have remained despite a pre-session exercise. To address these issues, participants were divided into two groups with counterbalanced task orders on Day 2, which reduced the potential influence of recall and learning effects on revision performance.

One advantage of our study is that we ensured consistency using standardised hardware, software, and delineation guidelines, reducing variability. However, this controlled approach may not fully reflect real-world clinical settings, which often involve diverse tools and practices. The time savings observed in this study need to be confirmed after clinical translation to evaluate their impact fairly. Additionally, the contouring software (ProKnow) was unfamiliar to all participants. Some advanced features of other commercial software, such as semi-automatic or automatic contouring tools, e.g., region-growing for lungs [31] or knowledge-based auto-segmentation [32], were unavailable. Lastly, the mouse-only setup, rather than a touch screen or stylus, may have limited the delineation speed [33]. Despite these limitations, our study provides a baseline assessment under unbiased, controlled conditions, highlighting the impact of DL models. Future studies may evaluate the implementation of these models in diverse clinical practices, such as different contouring software, to understand their broader applicability better.

On the other hand, this study has several strengths. First, it included a large group of paediatric radiation oncologists (11 + 1) with interest in the paediatric upper abdominal region. Second, the model evaluated is openly available and was developed using a paediatric dataset considerably larger than those available to most centres.

Additionally, the study provides a comprehensive understanding of the impact of integrating DL for delineation before manual revision. The findings can potentially assist clinics in optimising workload and resource allocation and improving treatment planning processes. For example, DL-based segmentation can support delineating structures as the lungs, liver, and pancreas, reducing time, accuracy, and inter-observer variability associated with manual delineation.

## Conclusions

This study evaluated a paediatric-specific DL-based auto-contouring model for flank irradiation in paediatric renal tumours. Our findings show the benefits of DL-assisted contouring with human revision in improving OAR delineation efficiency, accuracy, and consistency.

## CRediT authorship contribution statement

**Mianyong Ding:** Conceptualization, Data curation, Formal analysis, Investigation, Methodology, Software, Validation, Visualization, Writing – original draft, Writing – review & editing. **Matteo Maspero:** Conceptualization, Data curation, Formal analysis, Funding acquisition, Methodology, Resources, Software, Supervision, Validation, Writing – original draft, Writing – review & editing. **Semi Harrabi:** Data curation, Formal analysis, Writing – review & editing. **Emmanuel Jouglar:** Data curation, Formal analysis, Writing – review & editing. **Sabina Vennarini:** Data curation, Formal analysis, Writing – review & editing. **Timothy Spencer:** Data curation, Formal analysis, Writing – review & editing. **Britta Weber:** Data curation, Formal analysis, Writing – review & editing. **Henriette Magelssen:** Data curation, Formal analysis, Writing – review & editing. **Karen Van Beek:** Data curation, Formal analysis, Writing – review & editing. **Remus Stoica:** Data curation, Formal analysis, Writing – review & editing. **Simonetta Saldi:** Data curation, Formal analysis, Writing – review & editing. **Tom Boterberg:** Data curation, Formal analysis, Writing – review & editing. **Patrick Melchior:** Data curation, Formal analysis, Writing – review & editing. **Marry M. van den Heuvel-Eibrink:** Funding acquisition, Project administration, Resources, Supervision, Writing – review & editing. **Geert O. Janssens:** Conceptualization, Data curation, Formal analysis, Funding acquisition, Project administration, Resources, Supervision, Writing – review & editing.

## Funding

This project is supported by the HE/MSCA co-fund project no. 101081481, and the KiTZ-Maxima Twinning Program, which stimulates collaborations and joint projects between the Hopp Children’s Cancer Centre (KiTZ) in Heidelberg and the Princess Máxima Centre in Utrecht.

## Declaration of competing interest

The authors declare that they have no known competing financial interests or personal relationships that could have appeared to influence the work reported in this paper.

## Footnotes

1 The model can be accessed at: https://github.com/MMianyong/-PedAbdSeg-.
