# Supplementary material for: Impact of deep learning on CT-based organ-at-risk delineation for flank irradiation in paediatric renal tumours: a SIOP-RTSG radiotherapy committee study
Source: Clin Transl Radiat Oncol. 2025 Sep 19;56:101051. doi: 10.1016/j.ctro.2025.101051 (PMC12553021; doi:10.1016/j.ctro.2025.101051)

**Supplementary material 1**

This material summarizes the responses to 20 questions from the pre-workshop survey, completed by 12 participants.

**Q1. Total years of experience in Radiation Oncology (as a staff member)**


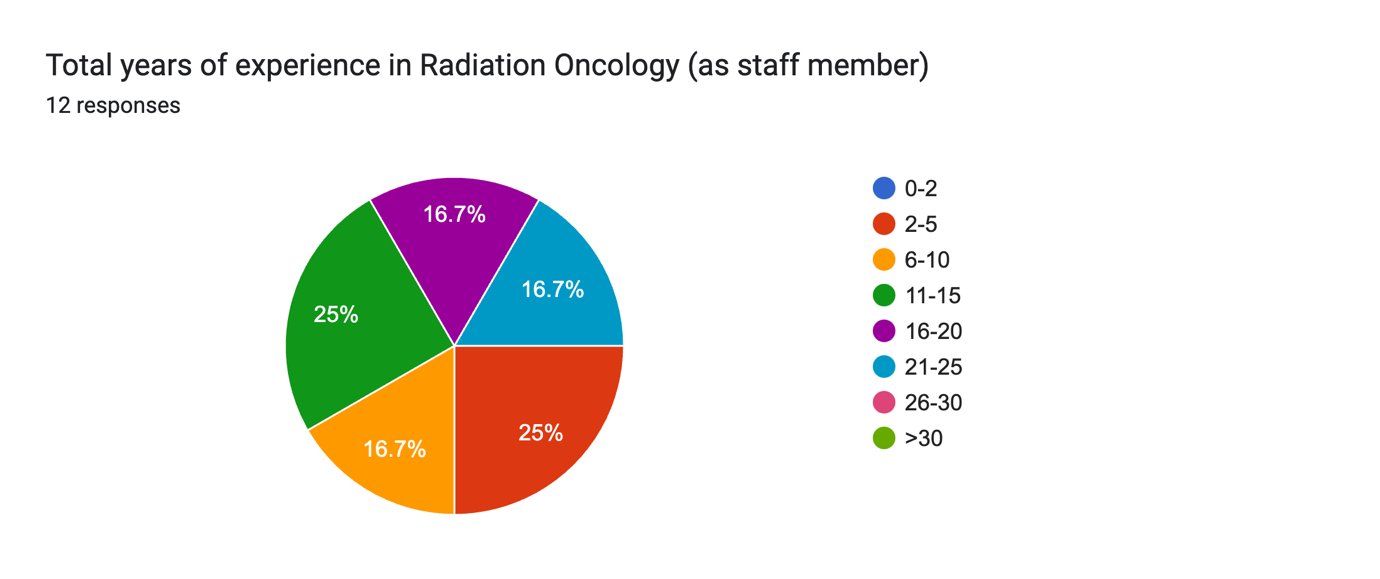


**Q2. (Approximate) annual number of paediatric patients with an indication for flank irradiation in your department**

**
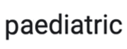
**
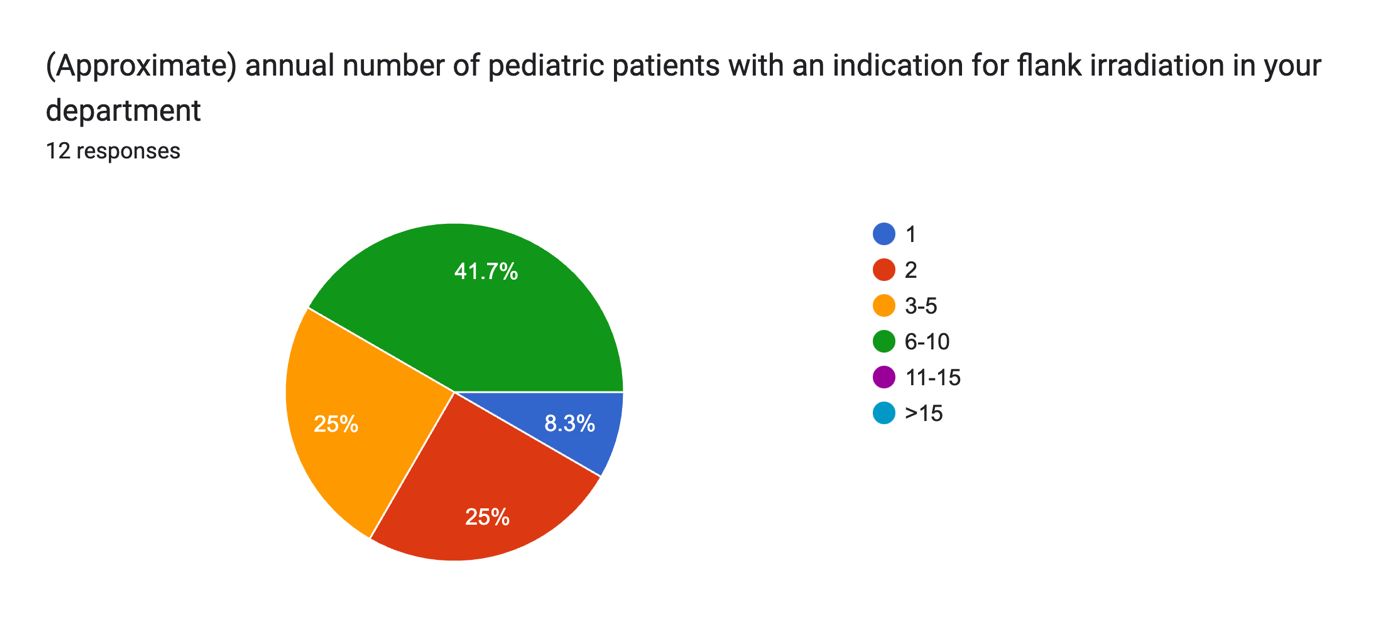


**Perspective and practice on/with AI models**

**Q3. How do you think artificial intelligence will impact radiotherapy?**


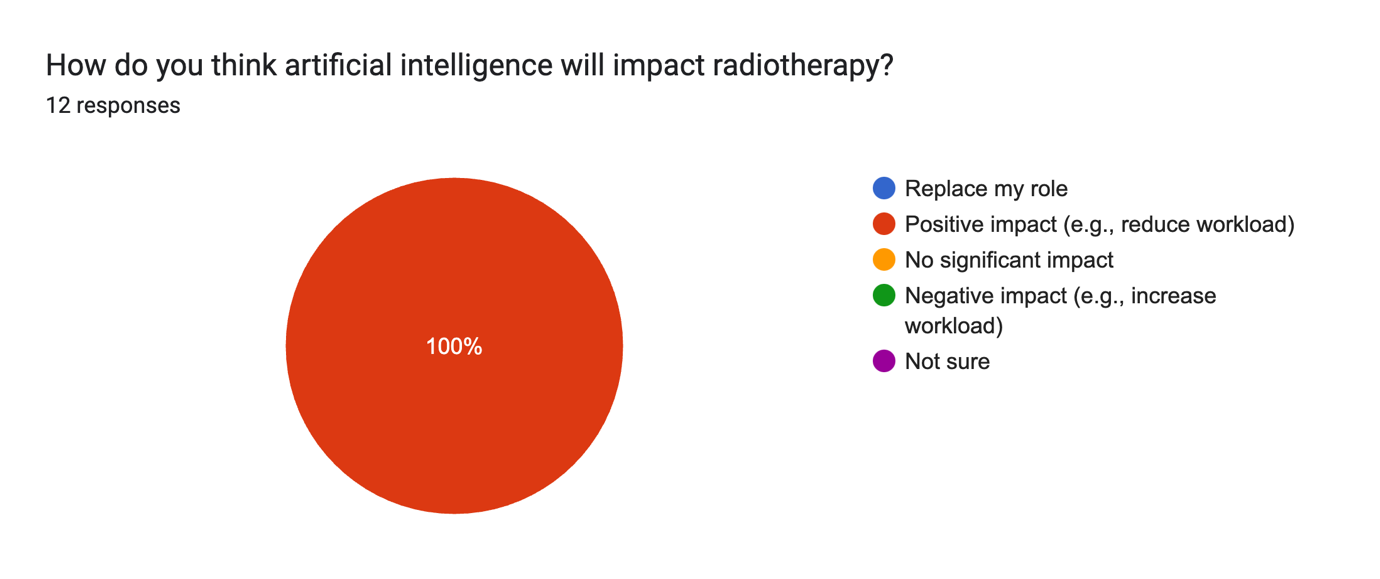


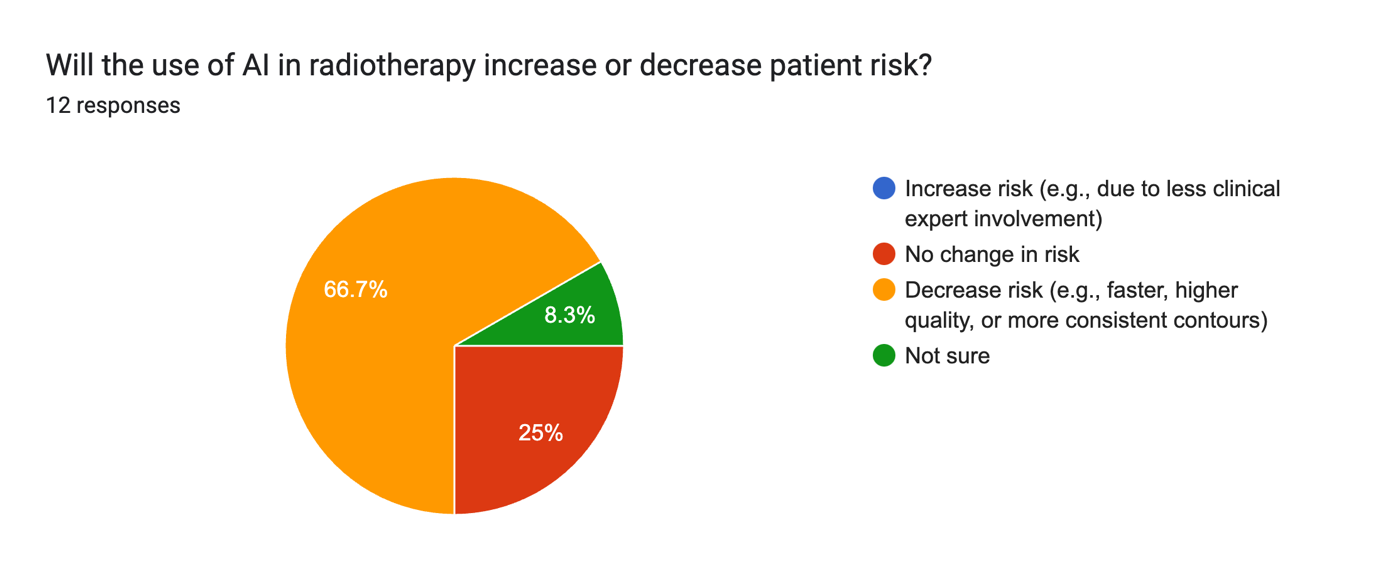
**Q4. Will the use of AI in radiotherapy increase or decrease patient risk?**

**Q5. What do you think is the potential risk of using auto-contouring AI models for OARs delineation?**

(12 responses, each bullet point shows one response)

- One of the risks is proceeding in the workflow of RT treatment without proper verification of the auto-contour which may lead to the delivery of treatment with a dose above the recommended constraints in OAR => more side effect
- Not enough clinical involvement in checking the AI contours
- Not supervising it critically
- No risk as long as AI is performed under human supervision
- Inexperienced staff will not recognize misplaced or wrong-contoured OARs
- The doctor relies more and more on AI and does not properly check the contours
- Trainees and newer consultants not accustomed to outlining OARs could become deskilled and less able to recognise errors or unusual anatomy
- I believe that using artificial intelligence in radiotherapy to define organs at risk allows for significant time savings and enhanced precision. AI systems are trained on vast datasets of medical cases, which enables them to recognize patterns and accurately identify critical structures in imaging. This extensive training helps the AI to provide consistent, high-quality results, eliminating much of the variability that can occur with manual contouring, by automating time-consuming tasks
- Huge anatomical differences in children according to age
- No specific risks that exceed risks of inter-individual OAR contouring
- I do not think there are risks but well a potential help in our work. All the work must then be reviewed by us as experts in the field
- Less attention from experts

**Q6**

**What do you think is the potential risk of using auto-contouring AI models for target delineation?**

(11 responses, each bullet point shows one response )

- One of the risks is proceeding in the workflow of RT treatment without proper verification of the auto-contour which may lead to the delivery of a treatment to a wider zone than it should => toxicity or missing the area at risk of relapse => more relapses
- Misinterpretation of the concepts behind contouring for instance tumorbed. Not enough clinical attention compared to previously when the clinical experts are making all the contours
- Not correct
- No risk as long as AI is performed under human supervision
- Same as above
- Doctors rely more and more on AI and do not properly check the clinical aspects
- As above, the risk of deskilling and becoming over-reliant on AI
- not enough data input before using clinically
- Ok for ‚standard cases with typical disease localisation - cautious in atypical growth patterns, rare constellations or in general scenarios the model wasn’t trained for)
- I do not think there are risks but well a potential help in our work. All the work must then be reviewed by us as experts in the field
- Faster and more consistent contours

**Q7. How would you rate your knowledge of AI, especially deep learning, for auto-contouring?**


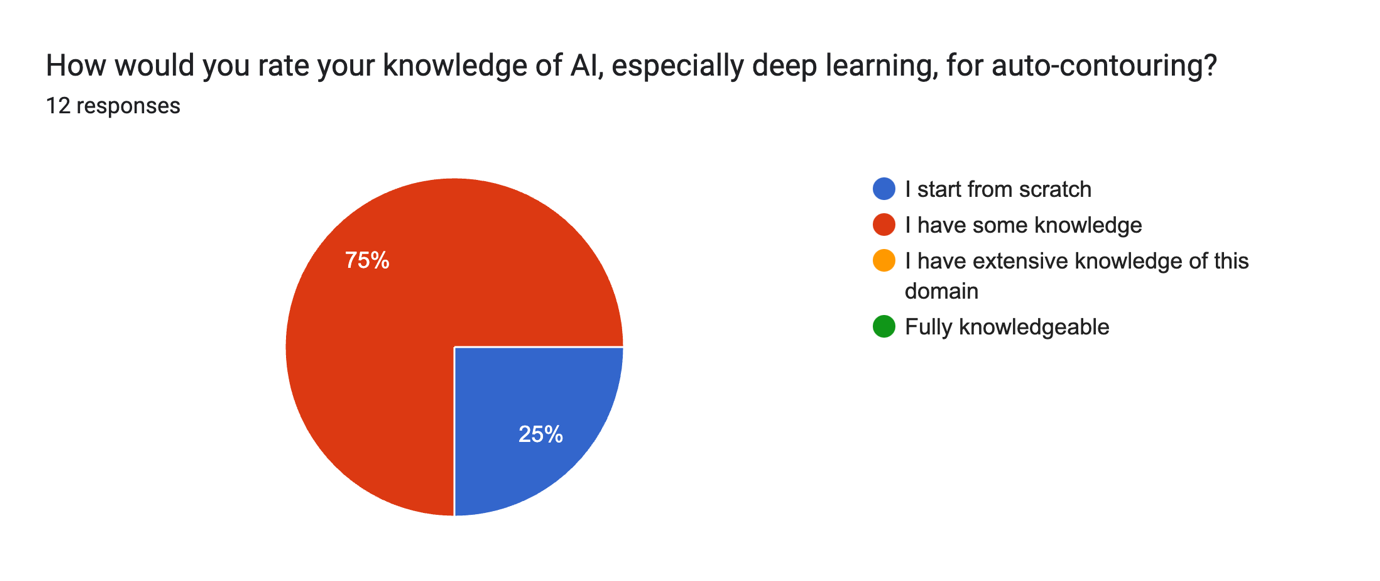


**Q8. Have you received formal training or guidance on using AI contouring tools?**


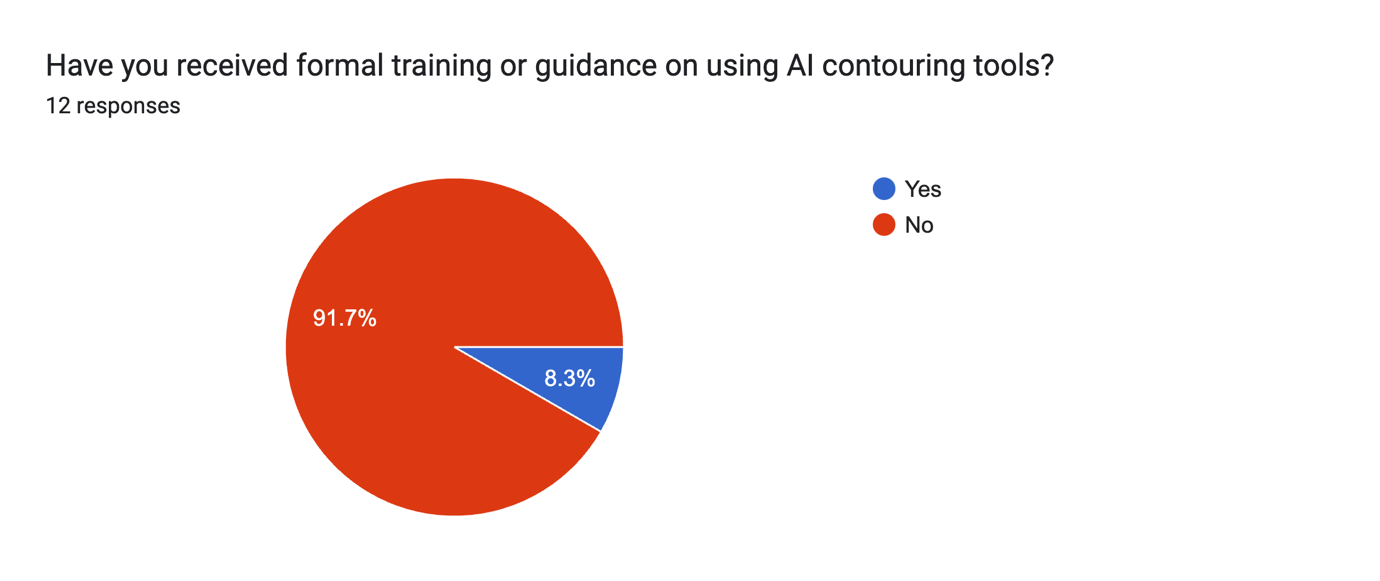


**Imaging, contouring software, and AI used in your department**


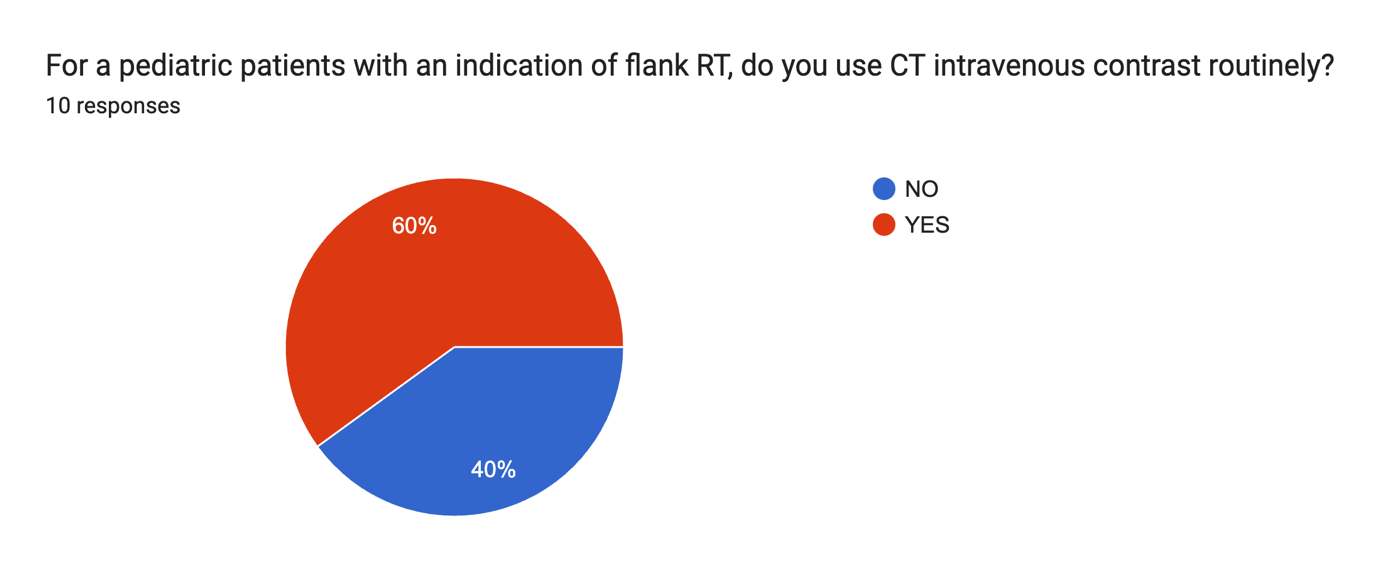
**Q9. For a paediatric patient with an indication of flank RT, do you use CT intravenous contrast routinely?**

**Q10. For a paediatric patient with an indication of flank RT, do you use CT oral contrast routinely?**


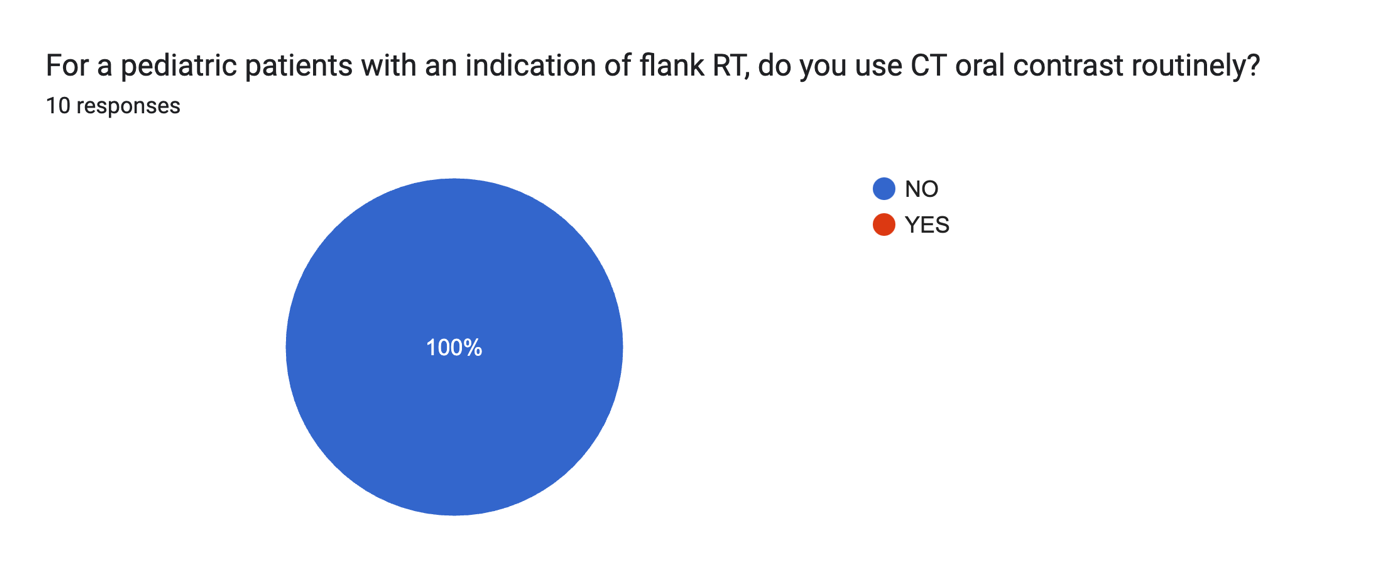


**Q11.** **For a pediatric patient with an indication of flank RT, do you perform a post-operative MRI for co-registration?**


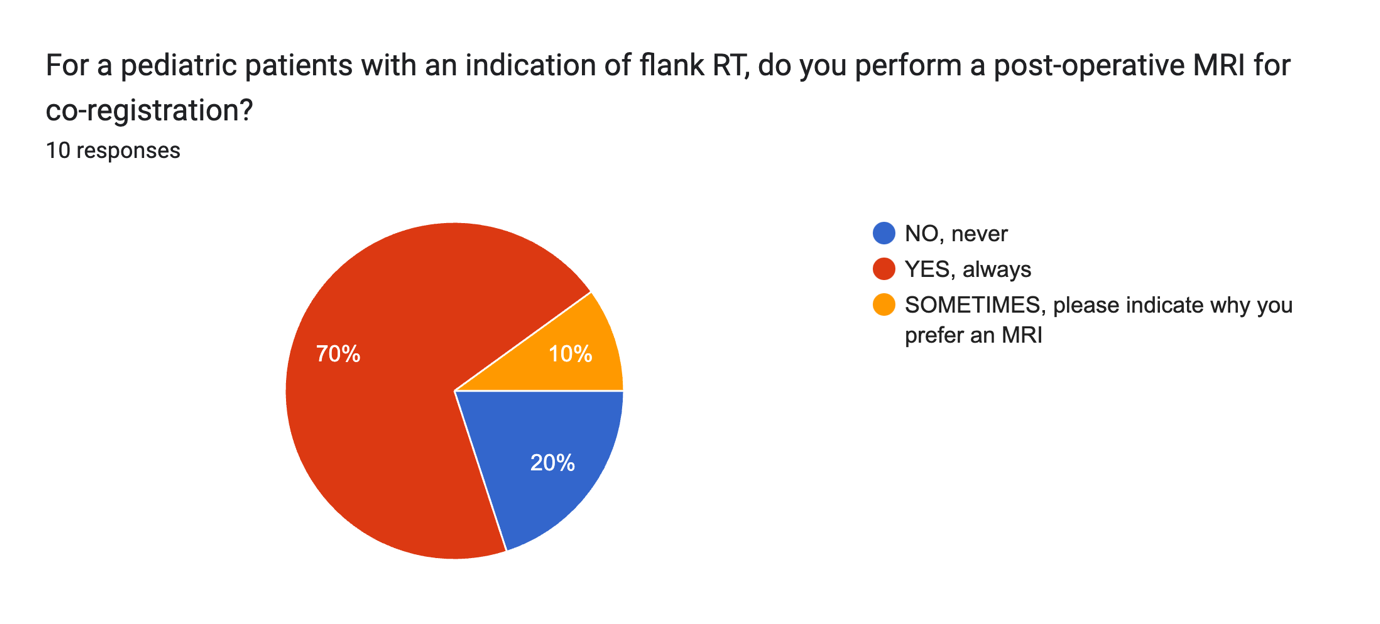


**Q12. If you use SOMETIMES, please indicate why you prefer an MRI**

(3 responses, each bullet point shows one response )

- To delineate pancreas
- A better view of potential residual disease
- NA

**Q13. Have you any previous experience with ProKnow (Elekta, AB), which is the software we will use during the workshop?**


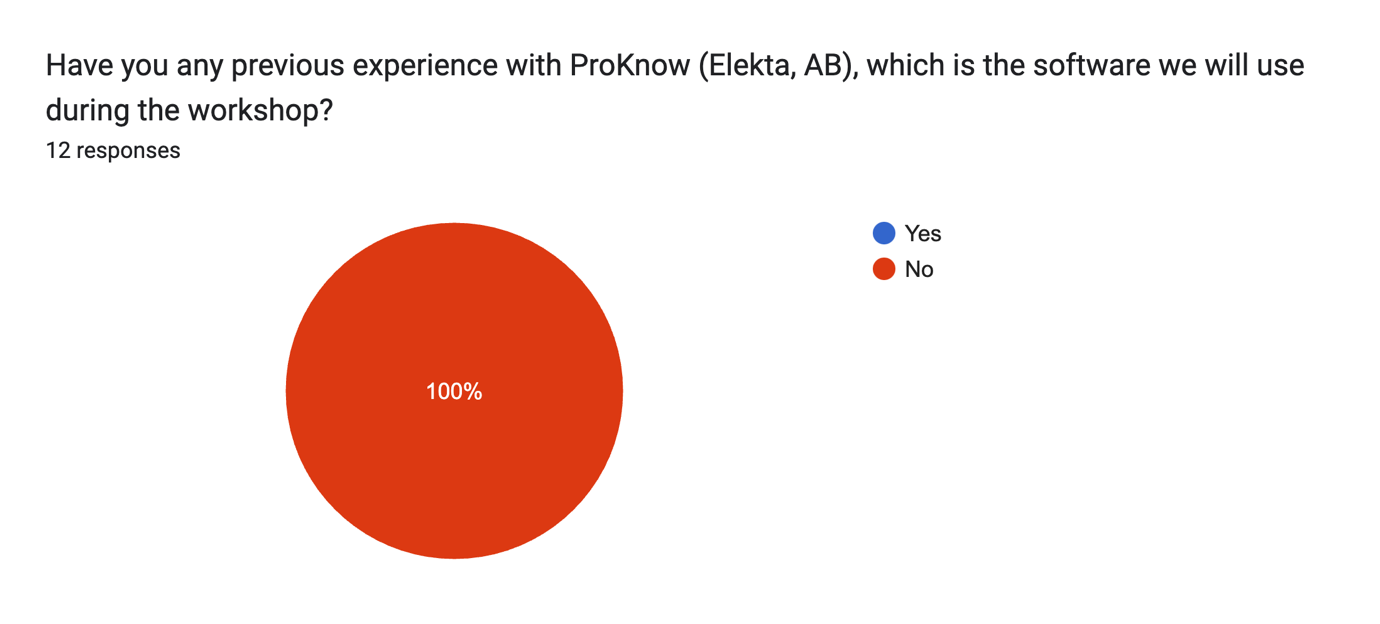


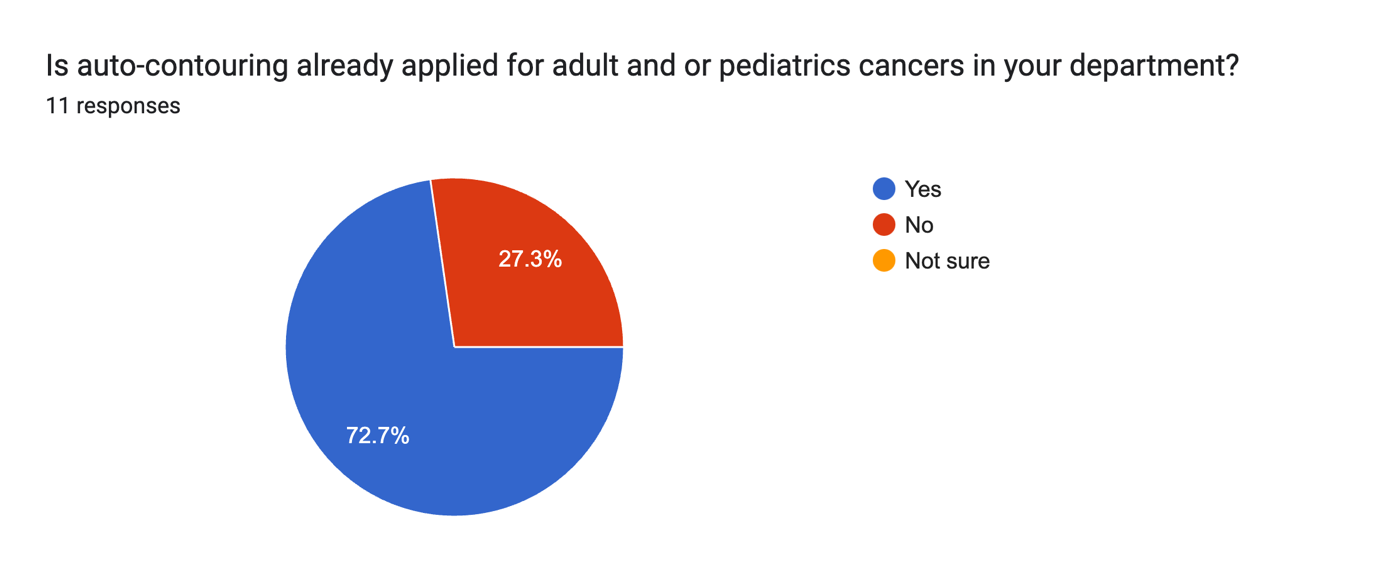
**Q14.** **Is auto-contouring already applied for adult and or paediatrics cancers in your department?**

**Q15. How do you perform OAR contouring in your department?**
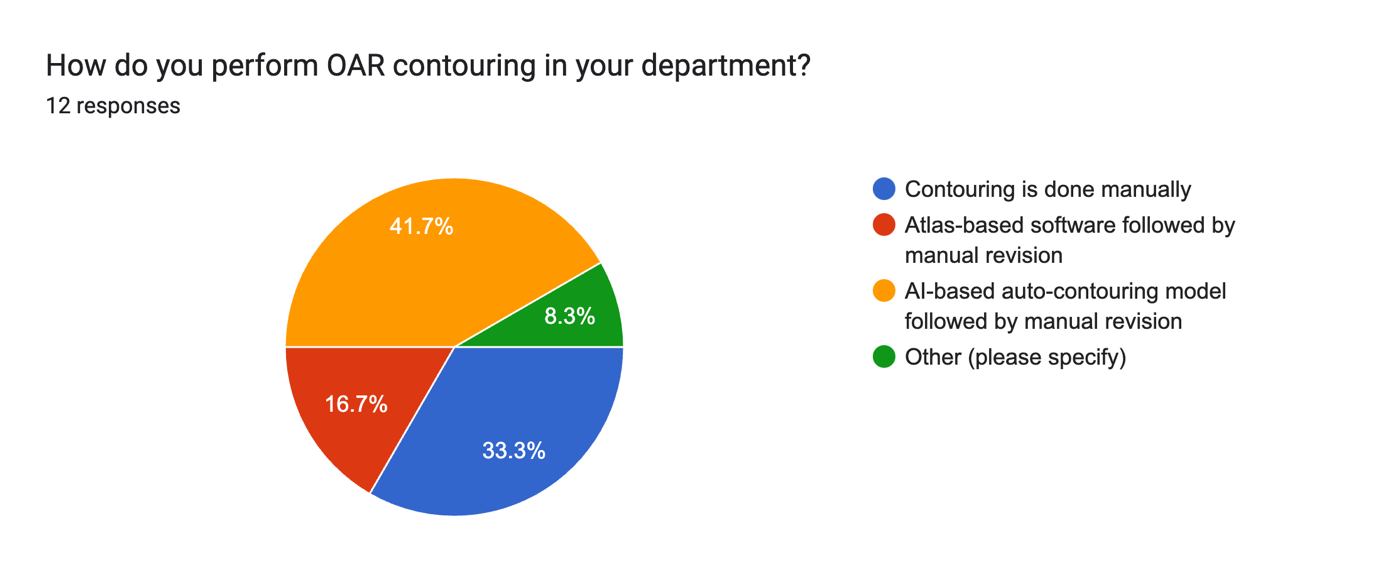


**Q16. Could you specify which contouring software(s) do you use?**

(9 responses, number in the parathness represent the number of reponses )

- Raystation(4)
- MIM (2)
- StringFilter(1)
- Eclipse (1)
- Elements (1)
- Limbus (1)
- Ethossystem (1)
- In house developed auto-contouring(1)
- Therapanacea (1)
- Both atlas- and AI-based depending on the region (1)


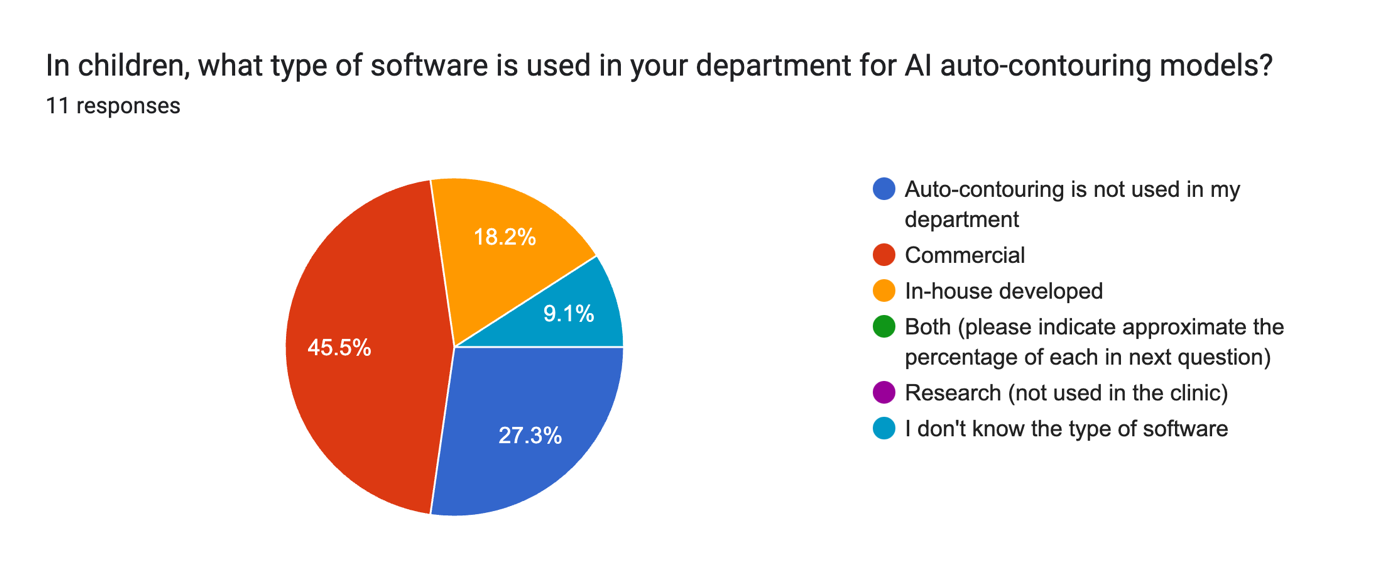
**Q17. In children, what type of software is used in your department for AI auto-contouring models?**

**Q18. In adults, what type of software is used in your department for AI auto-contouring models?**


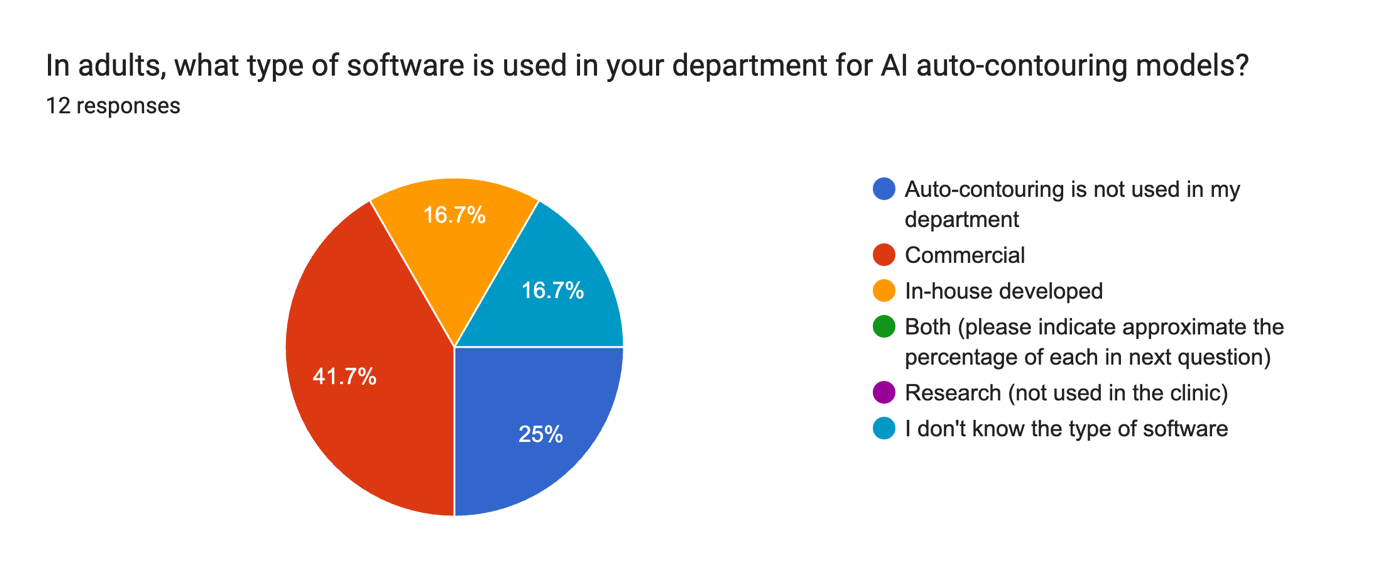


**Q19. In children, approximately what percentage of OAR contouring in your department is assisted by AI?**


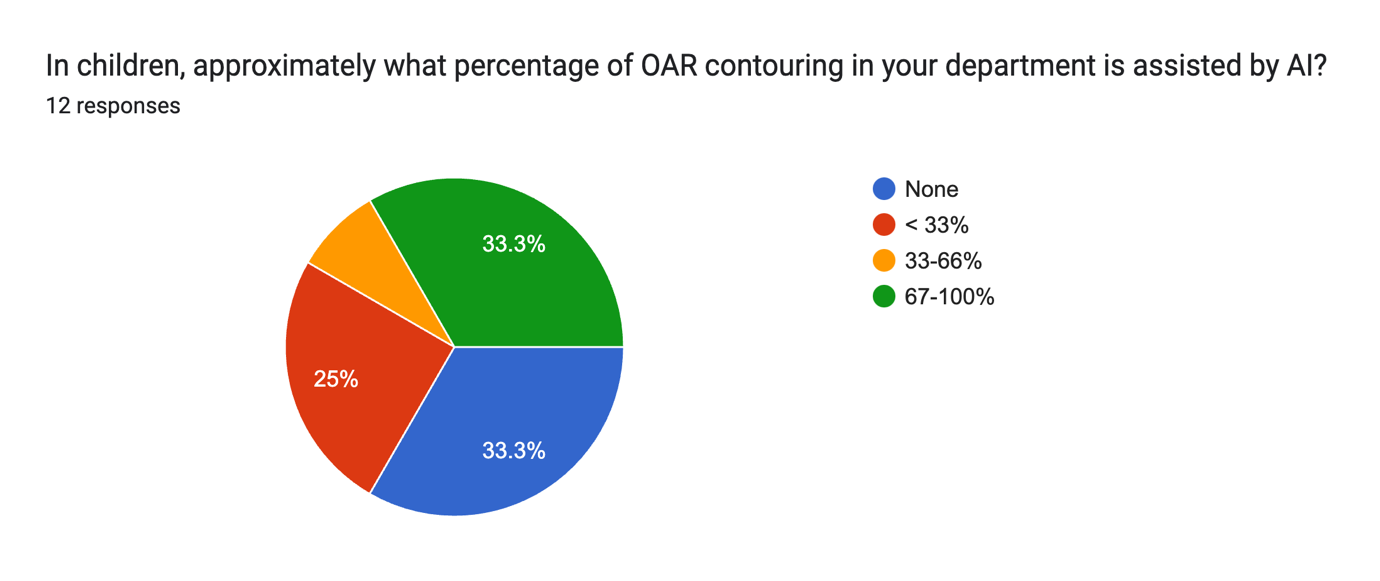


**Q20. Per pediatric patient, how much time does auto-contouring save on average?**


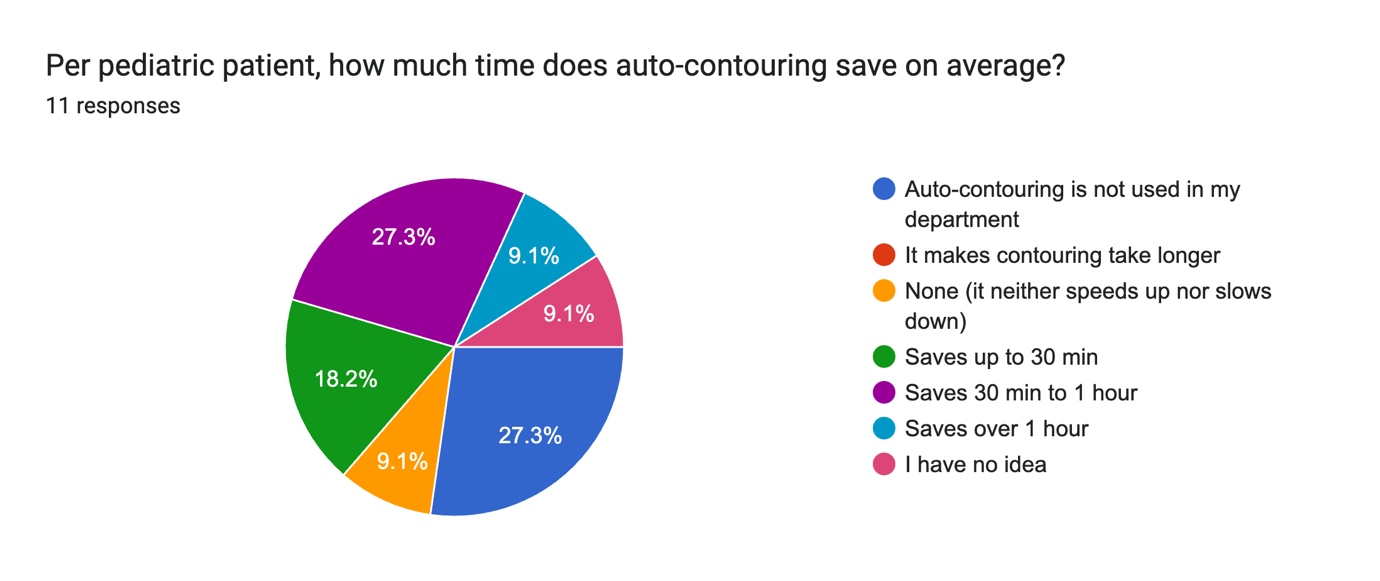


**Q21.** **For adults and children, do you inform patients when AI-based contouring software is used for the contouring of organs/structure at risk?**


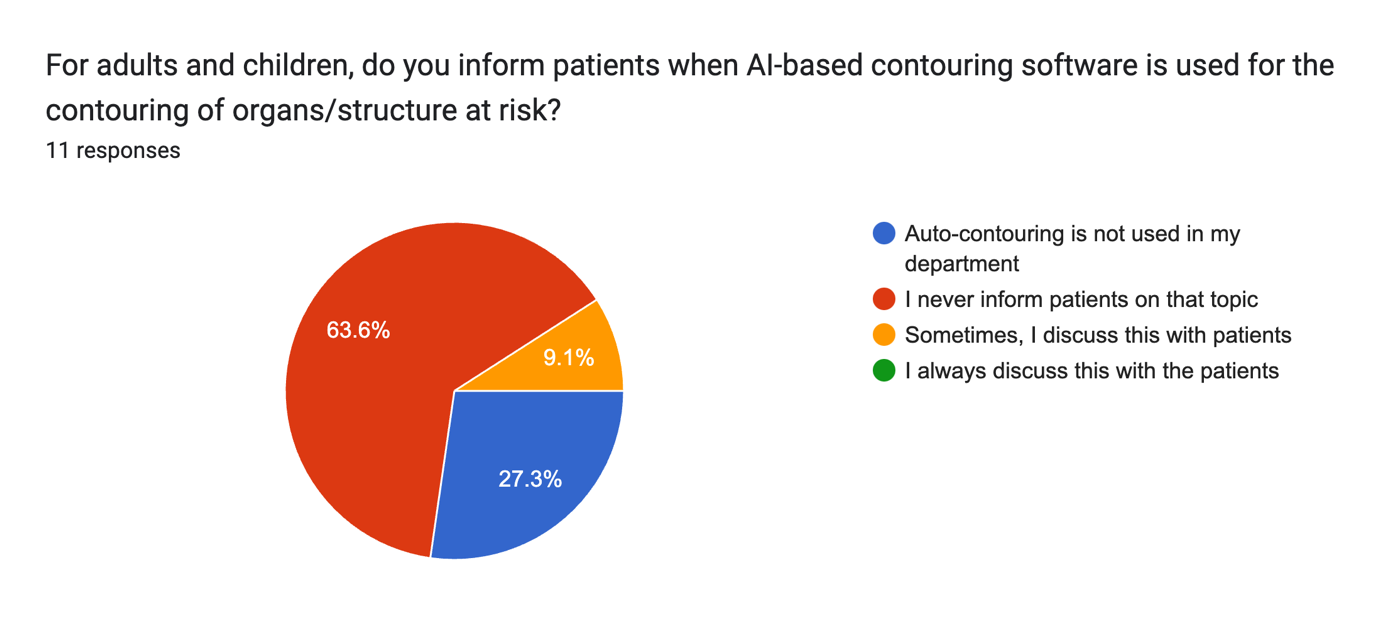

Supplement: Supplementary Data 1 [file mmc1.docx]
