# Supplementary material for: Impact of deep learning on CT-based organ-at-risk delineation for flank irradiation in paediatric renal tumours: a SIOP-RTSG radiotherapy committee study
Source: Clin Transl Radiat Oncol. 2025 Sep 19;56:101051. doi: 10.1016/j.ctro.2025.101051 (PMC12553021; doi:10.1016/j.ctro.2025.101051)
Supplement: Supplementary Data 2 [file mmc2.docx]

**Supplementary material 2**

This material provides the recommended window level and guidelines for each organs/strucutures-at-risk (OARs) to assist participants during the workshop. Additionally, delineations were performed on every 2–5 slices, utilizing the interpolate function in ProKnow for intermediate slices, followed by manual revisions.

**Supplementary Table 1.** Recommended window levels and guidelines for each OAR.

| Structures / OARs | Window | Level | Guidelines |
| --- | --- | --- | --- |
| Heart, Liver, Spleen, Kidneys | 200 | 20 | \|  \| \| --- \|  \|  \| \| --- \|   For each OARs, Delineate the whole organs. For the heart, start at the upper border where the vessels begin. For kidneys, only one kidney is present per patient. |
| Pancreas | 80 | 40 | Delineate the entire organ. |
| Lungs | 1700 | -500 | Delineate the entire organ. |
| Stomach-Bowel | 400 | 40 | Consider the bowel bag and include the stomach. Extend structures down to L4. |
