# Supplementary material for: Impact of deep learning on CT-based organ-at-risk delineation for flank irradiation in paediatric renal tumours: a SIOP-RTSG radiotherapy committee study
Source: Clin Transl Radiat Oncol. 2025 Sep 19;56:101051. doi: 10.1016/j.ctro.2025.101051 (PMC12553021; doi:10.1016/j.ctro.2025.101051)
Supplement: Supplementary Data 3 [file mmc3.docx]

**Supplementary Material 3**

This supplementary material provides additional analyses of delineation time, accuracy (Acc), and inter-observer variability (IOV) using various evaluation methods. It complements the main text by more comprehensive information.

First, the comparison between the STAPLE and single expert references shows the differences between the two using three metrics: Dice Similarity Coefficient (DSC), 95th Percentile Hausdorff Distance (HD95), and Mean Surface Distance (MSD).

Second, direct comparisons of time, accuracy, and IOV are presented for cases where the same patients were delineated twice by the same observers in both the manual and deep learning (DL)-based revision sessions. This controlled comparison highlights the performance of the DL-based model.

In the main text, STAPLE reference contours were generated using combined delineations from both sessions, which could potentially favor AI-generated contours For completeness, we also provide analyses using separate STAPLE reference contours generated independently for each session.

Lastly, a pairwise comparison approach is used to evaluate IOV. This method calculates variability across all pairs of delineations made by different observers.

The following tables/figures illustrate these analyses

**STAPLE Reference vs. Single Expert’s Reference**

- **Supplementary Table 2**. Comparison between the STAPLE reference and the single expert reference.

**Direct comparison of time, accuracy, and IOV**

- **Supplementary Figure 1.** Direct comparison of delineation times between manual delineation and DL-based revision sessions for the same patients delineated by the same participants.
- **Supplementary Figure 2.** Direct comparison of accuracy and IOV between manual delineation and DL-based revision sessions for the same patients delineated by the same participants, using single-expert reference contours.
- **Supplementary Figure 3.** Direct comparison of accuracy and IOV between manual delineation and DL-based revision sessions for the same patients delineated by the same participants, using STAPLE reference contours.

**Session-specific STAPLE references**

- **Supplementary Figure 4.** Comparison of accuracy and IOV between manual delineation and DL-based revision sessions using STAPLE reference contours generated separately for each session.

**Pairwise comparison to evaluate inter-observer variability (IOV)**

- **Supplementary Figure 5.** Pairwise comparison of inter-observer variability (IOV) between manual and DL-based revision sessions, based on included contours.
- **Supplementary Figure 6.** Pairwise comparison of inter-observer variability (IOV) between manual and DL-based revision sessions for the same patients delineated by the same observer across both sessions.

**STAPLE Reference vs. Single Expert’s Reference**

The single expert reference has the advantage of no time constraints and access to MRI, potentially resulting in higher precision. Additionally, it is free from the influence of DL-based delineations. However, it may be subject to individual biases, as it reflects the expertise and judgment of a single participant.

In contrast, the STAPLE reference generates a consensus based on delineations from all participants across both sessions, which reduces individual biases. However, it can still be influenced by the varying expertise levels of the participants. Furthermore, since participants did not have access to MRI and were working under time constraints, the precision of the STAPLE reference may be lower. Additionally, the inclusion of DL-based revision delineations in the STAPLE generation introduces potential bias from the deep learning model itself.

To assess the differences between the STAPLE reference and the single expert reference, Supplementary Table 2 compares the two using metrics such as Dice Similarity Coefficient (DSC), HD95, and Mean Surface Distance (MSD). Structures like the kidney, liver, and lung showed relatively high similarity between the two references, whereas structures like the pancreas demonstrated lower similarity.

**Supplementary Table 2**. Comparison between the STAPLE reference and the single expert reference.

| OARs | DSC | HD95(mm) | MSD (mm) |
| --- | --- | --- | --- |
| Heart | 0.93±0.01 | 4.0±0.76 | 0.97±0.08 |
| Kidney | 0.96±0.01 | 1.26±0.36 | 0.49±0.1 |
| Kidney | 0.97±0.0 | 1.12±0.35 | 0.38±0.06 |
| Liver | 0.97±0.0 | 1.55±0.37 | 0.56±0.09 |
| Lung-L | 0.96±0.01 | 1.36±0.44 | 0.51±0.17 |
| Lung-R | 0.97±0.01 | 1.36±0.56 | 0.48±0.18 |
| Pancreas | 0.82±0.04 | 2.68±1.34 | 0.96±0.37 |
| Spleen | 0.94±0.01 | 2.09±0.79 | 0.61±0.08 |
| Stomach-Bowel | 0.94±0.01 | 4.6±1.74 | 1.01±0.23 |

**Direct comparison of time, accuracy, and IOV**

To provide a more direct comparison, we analyzed cases where the same participants performed both manual delineations and DL-based revisions on the same patients. Cases that were delineated only in the DL-based revision session were excluded from this analysis. A paired Wilcoxon signed-rank test was used to compare delineation times, ensuring a controlled and straightforward comparison.

Although the reduced sample size in the DL-based revision session lowered statistical power and limited the generalizability of the findings, the results regarding time comparsion (Supplementary Figure 1) were consistent with those reported in Figure 2 of the main text. Except for the stomach-bowel, all OARs showed significant time savings in the DL-based session. When all OARs were analyzed together, the total delineation time decreased from 62.53 minutes to 29.66 minutes, representing an overall time savings of 52.6%.

A similar conclusion was observed for accuracy and IOV through direct comparisons using a single expert as the reference (Supplementary Figure 2). Across all OARs except the stomach-bowel, at least two metrics showed significant improvements in accuracy for the DL-based revision session. Direct comparison also revealed significantly reduced IOV in at least two metrics for certain OARs, such as the liver and lungs. For the pancreas, the standard deviation decreased by at least fourfold, although this result did not reach statistical significance.

When STAPLE was used as the reference (Supplementary Figure 3), similar patterns were observed. Except for the stomach-bowel, all OARs showed significantly improved accuracy in at least one metric. For IOV, significant reductions were found in at least two metrics for most OARs, except for the kidney and stomach-bowel.

**Supplementary Figure 1.** Direct comparison of delineation times between manual delineation and DL-based revision sessions for the same patients delineated by the same participants. P-values were calculated using the Wilcoxon signed-rank test.


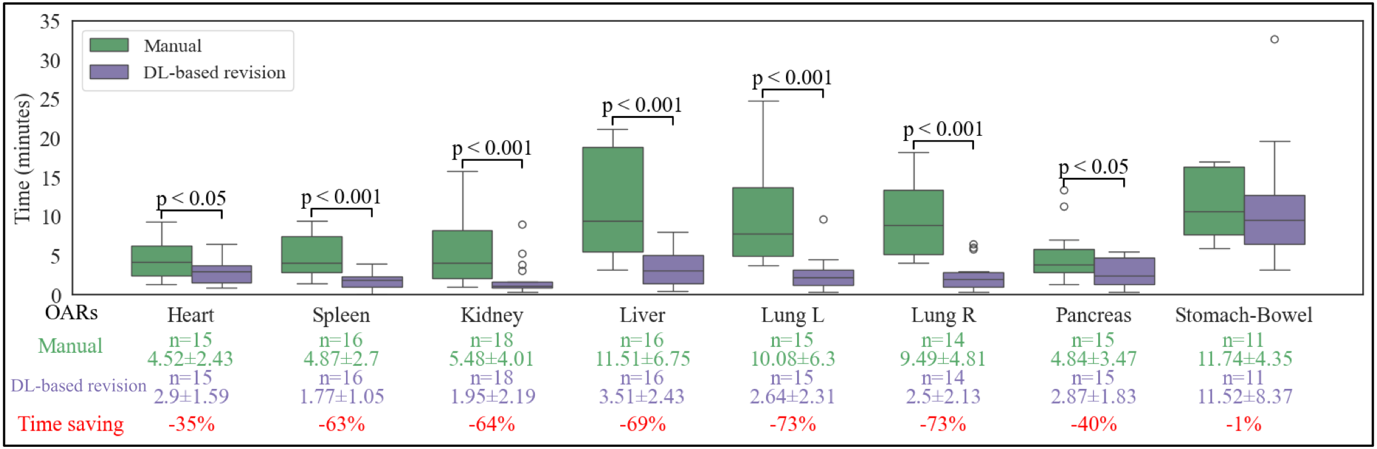


**Supplementary Figure 2.** Direct comparison of accuracy and IOV between manual delineation and DL-based revision sessions for the same patients delineated by the same participants, using single-expert reference contours. P-values were calculated using the Wilcoxon signed-rank test.

**Supplementary Figure 3.** Direct comparison of accuracy and IOV between manual delineation and DL-based revision sessions for the same patients delineated by the same participants, using STAPLE reference contours.


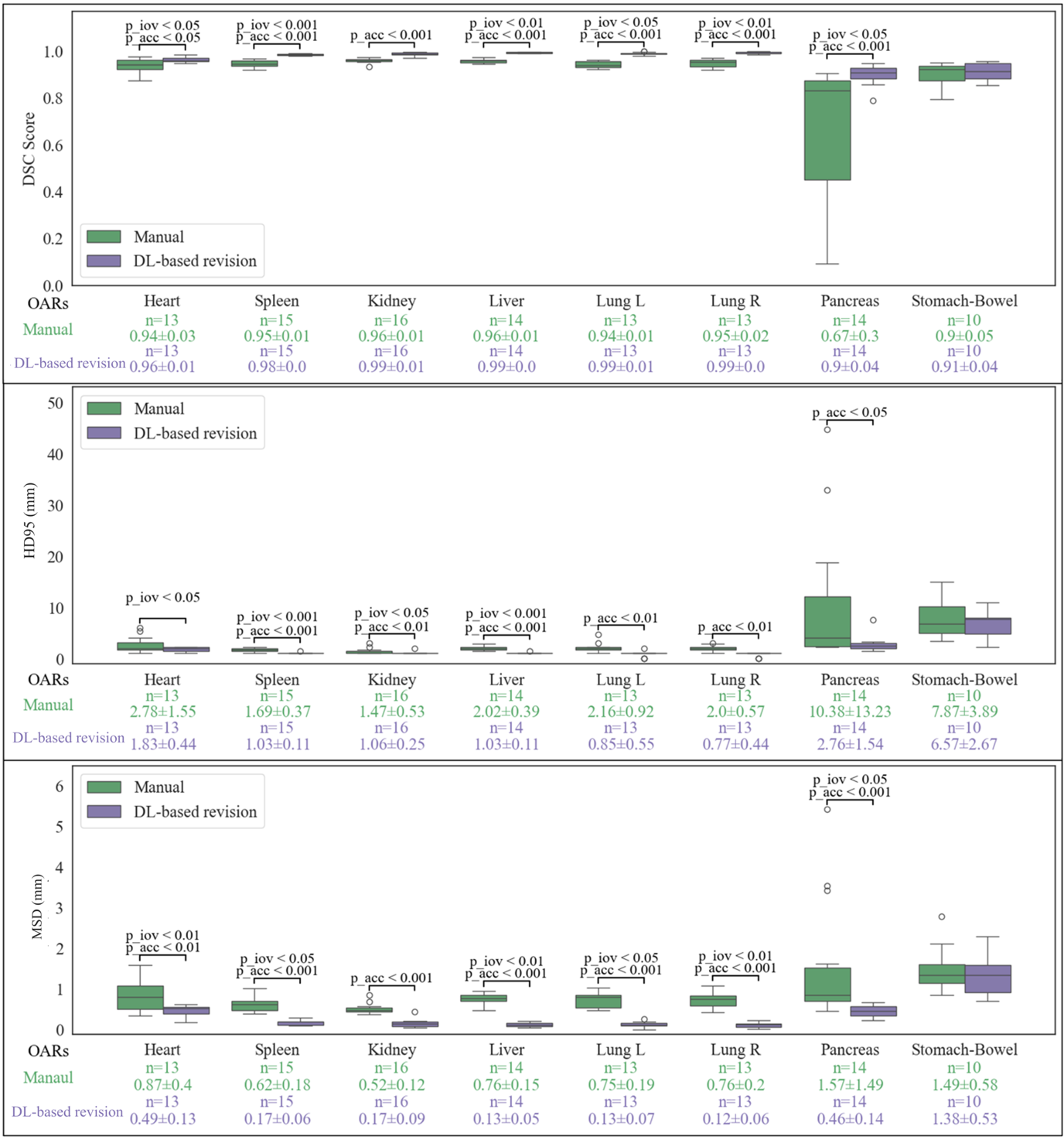


**Session-specific STAPLE references**

The STAPLE reference was generated in two ways. The combined STAPLE was created using delineations from both sessions, resulting in a single reference for each patient. This ensured consistency for direct comparison, as reported in the main text, but it may have been influenced by AI-based delineations, potentially skewing the results.

Separate STAPLE references were generated for each session, using only delineations from that specific session. This ensured that manual delineation references were unaffected by AI-based revisions but made accuracy comparisons more challenging. For each organ-at-risk (OAR) of each patient in each session, OARs with fewer than three delineations were excluded to maintain reliability.

The results from the separate STAPLE references (Supplementary Figure 4) were consistent with those from the combined STAPLE in showing improved accuracy and reduced inter-observer variability (IOV). The only differences were that IOV for kidney delineations in Dice Similarity Coefficient (DSC) and spleen in HD95 were observed in the separate STAPLE but not in the combined STAPLE.

**Supplementary Figure 4.** Comparison of accuracy and IOV between manual delineation and DL-based revision sessions using STAPLE reference contours generated separately for each session.


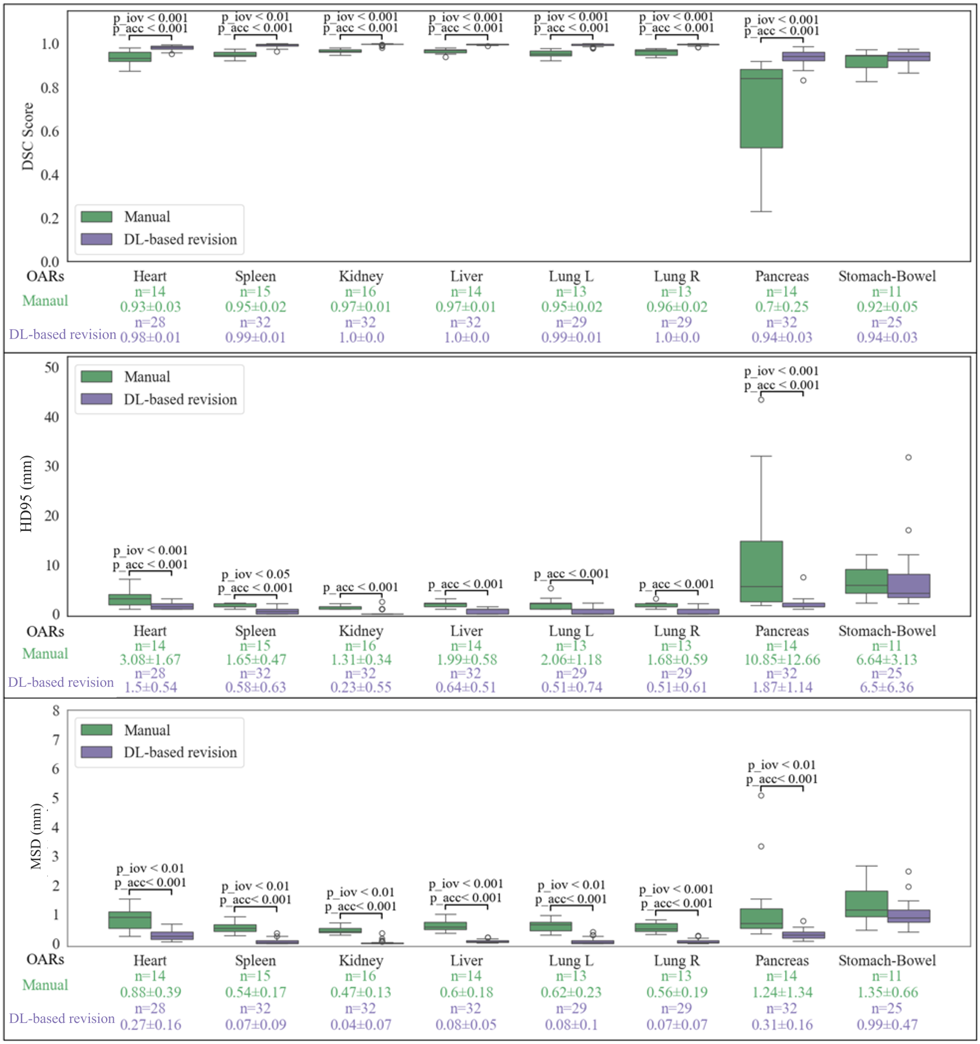


**Pairwise comparison to evaluate inter-observer variability (IOV)**

Pairwise comparisons of DSC, HD95, and MSD were conducted to evaluate IOV, a method commonly employed in similar studies to investigate the IOV. These metrics do not require a reference delineation, where higher DSC, lower HD95, and lower MSD indicate smaller differences and more consistent delineations between observers.

The pairwise calculations were performed for all pairs of contours generated by different observers for the same patient within each session. OARs from patients with fewer than two delineations were excluded from the analysis. The Wilcoxon rank-sum test was used to compare IOV between the two sessions, as shown in Supplementary Figure 5. Additionally, to directly compare the same patients delineated by the same observers across the two sessions, the Wilcoxon signed-rank test was applied, with results presented in Supplementary Figure 6.

The pairwise comparisons revealed that the DL-based revision significantly reduced IOV across all OARs, as shown in both plots. These findings reinforce the conclusion that DL-based auto-contouring with manual revision improves consistency in delineation among observers.

**Supplementary Figure 5.** Pairwise comparison of inter-observer variability (IOV) between manual and DL-based revision sessions, based on included contours. Statistical significance was assessed using the Wilcoxon rank-sum test.

**Supplementary Figure 6.** Pairwise comparison of IOV between manual and DL-based revision sessions for the same patients delineated by the same observer across both sessions. Statistical significance was assessed using the Wilcoxon signed-rank test.
