# Supplementary material for: Impact of deep learning on CT-based organ-at-risk delineation for flank irradiation in paediatric renal tumours: a SIOP-RTSG radiotherapy committee study
Source: Clin Transl Radiat Oncol. 2025 Sep 19;56:101051. doi: 10.1016/j.ctro.2025.101051 (PMC12553021; doi:10.1016/j.ctro.2025.101051)
Supplement: Supplementary Data 4 [file mmc4.docx]

**Supplementary Material 4**

**3D surface variation analysis**

We performed an additional 3D surface variation analysis using a method modified from the original reference [1]. Instead of using the median contour from each session (as in the original paper), we used a single STAPLE-generated reference contour derived from all delineations across both the manual and DL-revision sessions. This ensured consistency between the two sessions, as the median contour would otherwise differ between them. Using the same STAPLE reference reduces variability introduced by differences in the reference contour.

To calculate the global 3D surface variation:

1. For each surface point on the STAPLE contour of each OAR for each patient, we calculated the shortest distance from that point to the surface of each participant’s contour. The standard deviation (SD) of these distances across participants was then computed.
2. The mean of these per-point SD values was taken to obtain the SD for that OAR in that patient.
3. Finally, these patient-level OAR SD values were averaged across all patients to yield the global SD for that OAR.

Supplementary Table 3 presents the results of the 3D surface variation analysis. All OARs showed reduced variation in the DL-revision session compared with the manual session. The pancreas shows the largest reduction in inter-observer variability, consistent with the findings reported in the main manuscript.

**Supplementary Table 3.** Comparison of 3D surface variation between the manual and DL-revision sessions

| Organ | IOV_SD_mm (Manual) | IOV_SD_mm (DL revision) |
| --- | --- | --- |
| Heart | 1.24 ± 0.19 | 0.45 ± 0.09 |
| Kidney | 0.45 ± 0.07 | 0.06 ± 0.04 |
| Liver | 0.79 ± 0.09 | 0.13 ± 0.02 |
| Lung-L | 0.67 ± 0.15 | 0.13 ± 0.03 |
| Lung-R | 0.66 ± 0.07 | 0.09 ± 0.03 |
| Pancreas | 7.28 ± 5.57 | 0.67 ± 0.28 |
| Spleen | 0.55 ± 0.15 | 0.08 ± 0.05 |
| Stomach-Bowel | 2.92 ± 0.21 | 1.68 ± 0.06 |

**Time savings at the individual observer level**

Here we present the mean time savings for each participant (Supplementary Figure 7), which illustrates that time savings were not consistent across observers. No clear pattern emerged indicating that participants with more years of experience achieved less time savings, or that those with fewer years achieved greater time savings. It should be noted, however, that the number of participants in each experience group was small—for example, the 21–25 years group included only one participant—so these results are based on limited samples and cannot be generalized.

In addition, session order may have acted as a confounding factor for time savings. As shown in Supplementary Figure 7, participants in Group 2 tended to show lower time savings, likely because they completed the DL-revision session first and the manual session second. On the second day, participants may have been more familiar with the cases and anatomical structures, which could have enabled them to complete the manual session more quickly, thereby reducing the observed time savings.

**
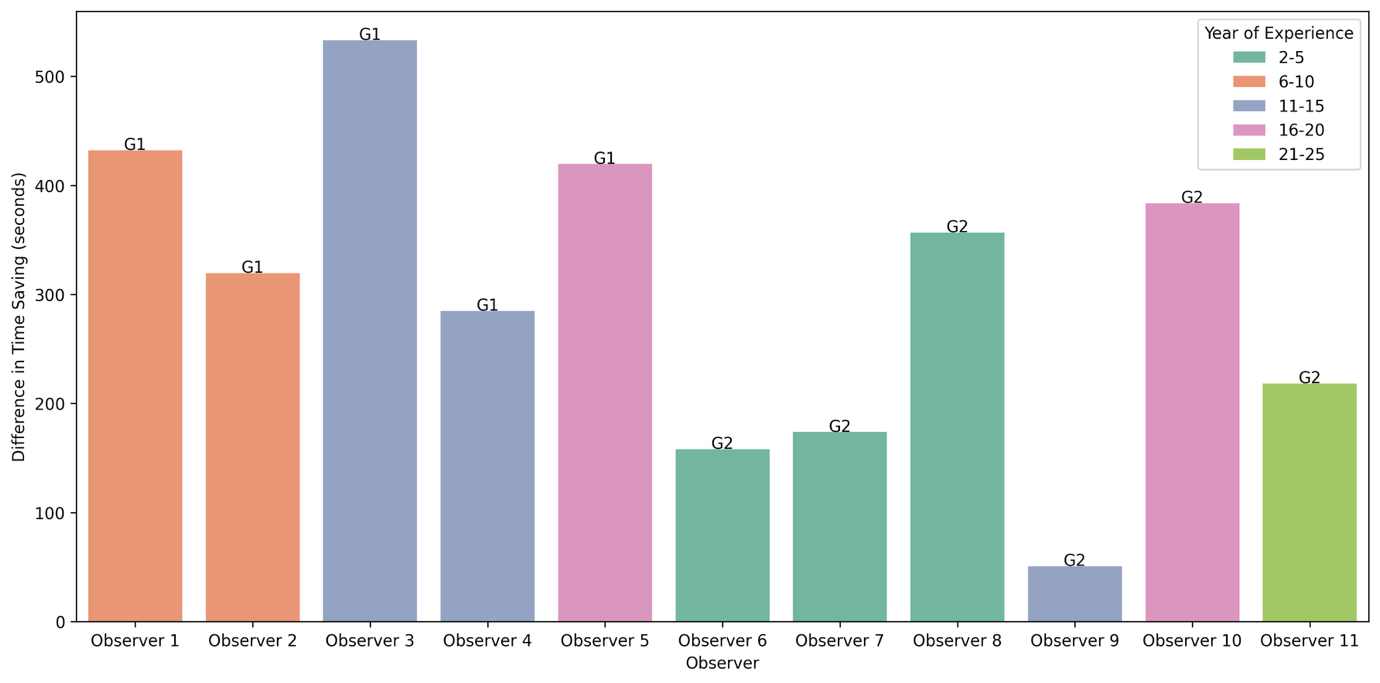
**

**Supplementary Figure 7.** Mean time savings per participant, expressed as the difference between the average delineation time per organ in the manual session and the DL-revision session. Colors indicate participants’ years of experience. Group 1 (G1) completed the manual session on the first day, followed by the DL-revision session on the second day, whereas Group 2 (G2) completed the sessions in the reverse order.

**References:**

[1] Brouwer CL, Steenbakkers RJ, van den Heuvel E, Duppen JC, Navran A, Bijl HP, Chouvalova O, Burlage FR, Meertens H, Langendijk JA, van't Veld AA. 3D Variation in delineation of head and neck organs at risk. Radiation Oncology. 2012 Mar 13;7(1):32.
